# Sub-Lethal Concentrations of Graphene Oxide Trigger Acute-Phase Response and Impairment of Phase-I Xenobiotic Metabolism in Upcyte^®^ Hepatocytes

**DOI:** 10.3389/fbioe.2022.867728

**Published:** 2022-05-19

**Authors:** A. Romaldini, R. Spanò, F. Catalano, F. Villa, A. Poggi, S. Sabella

**Affiliations:** ^1^ D3 PharmaChemistry, Istituto Italiano di Tecnologia, Genoa, Italy; ^2^ Electron Microscopy Facility, Istituto Italiano di Tecnologia, Genoa, Italy; ^3^ Unit of Molecular Oncology and Angiogenesis, IRCCS Ospedale Policlinico San Martino, Genoa, Italy

**Keywords:** cytotoxicity, apoptosis, cytochrome P450, acute phase proteins, nanomaterial induced hepatotoxicity, liver in vitro model, graphene oxide, hepatocytes

## Abstract

The impact of graphene oxide on hepatic functional cells represents a crucial evaluation step for its potential application in nanomedicine. Primary human hepatocytes are the gold standard for studying drug toxicity and metabolism; however, current technical limitations may slow down the large-scale diffusion of this cellular tool for *in vitro* investigations. To assess the potential hepatotoxicity of graphene oxide, we propose an alternative cell model, the second-generation upcyte^®^ hepatocytes, which show metabolic and functional profiles akin to primary human hepatocytes. Cells were acutely exposed to sub-lethal concentrations of graphene oxide (≤80 μg/ml) for 24 h and stress-related cell responses (such as apoptosis, oxidative stress, and inflammatory response) were evaluated, along with a broad investigation of graphene oxide impact on specialized hepatic functions. Results show a mild activation of early apoptosis but not oxidative stress or inflammatory response in our cell model. Notably, while graphene oxide clearly impacted phase-I drug-metabolism enzymes (e.g., CYP3A4, CYP2C9) through the inhibition of gene expression and metabolic activity, conversely, no effect was observed for phase-II enzyme GST and phase-III efflux transporter ABCG2. The GO-induced impairment of CYP3A4 occurs concomitantly with the activation of an early acute-phase response, characterized by altered levels of gene expression and protein production of relevant acute-phase proteins (i.e., CRP, Albumin, TFR, TTR). These data suggest that graphene oxide induces an acute phase response, which is in line with recent *in vivo* findings. In conclusion, upcyte^®^ hepatocytes appear a reliable *in vitro* model for assessing nanomaterial-induced hepatotoxicity, specifically showing that sub-lethal doses of graphene oxide have a negative impact on the specialized hepatic functions of these cells. The impairment of the cytochrome P450 system, along with the activation of an acute-phase response, may suggest potential detrimental consequences for human health, as altered detoxification from xenobiotics and drugs.

## Introduction

Graphene is a carbon-based material consisting of a single layer of sp^2^-hybridized carbon atoms arranged in a two-dimensional (2D) honeycomb-shaped lattice (Novoselov 2004). In the last decade, graphene and its derivatives have raised a huge interest in many industrial fields (e.g., electronics, energy storage, or thermal isolation) due to their peculiar physical-chemical characteristics.

In particular, graphene oxide (GO) is a promising nanomaterial for nanomedicine (Liu et al., 2013; Zhao et al., 2017; Dasari Shareena et al., 2018), including drug and gene delivery, bioimaging, and tissue engineering. GO is commonly obtained via graphite oxidation and its chemical structure is characterized by the presence of highly reactive oxygenated functional groups, such as epoxy, hydroxyl, and carboxyl groups (Loh et al., 2010; Chen et al., 2012). These functionalities make GO water-soluble and easily conjugable to bio-macromolecules or small ligands. Therefore, based on such intrinsic properties, GO can be considered a suitable candidate for loading drugs or other bioactive molecules and/or for acting as vehicle system of drugs to treat, for instance, cancer (Liu et al., 2021) or pathologies affecting the brain in the paediatric age (Cellot et al., 2021).

However, the technological boost of GO raises concerns related to its safety for human use (Fadeel et al., 2018). The innovation process of developing GO as a novel drug requires the application of toxicology tools to predict toxicity at an early stage of the material design and development, thus offering a rapid safety screening for many combinations of GO-based materials. This approach can allow for the quick identification of dose-limiting toxicities (e.g., sub-lethal doses), along with the material impact on organ-specific functionalities (e.g., hepatotoxicity). Furthermore, the application of *in vitro* models accounting for human body compartments (e.g., gastrointestinal tract, lung, liver, spleen, and so forth) may be relevant due to their central role in drug pharmacokinetics (i.e., administration, distribution, metabolism, and elimination (Fadeel et al., 2018)).

*In vivo* studies on bio-distribution reported that, after entering the bloodstream (through primary or secondary routes), GO may accumulate in many organs, including the liver, where interactions with hepatic functional cells may take place (Chen et al., 2015; Jasim et al., 2015; Mao et al., 2015; Wu et al., 2020). In particular, the accumulation of GO was also evidenced across the hepatic lobules in GO-exposed mice, revealing a preferential accumulation at the peripheral part of the lobules than the internal zones around the central vein (Wu et al., 2020). Moreover, although the clearance via faeces and/or urine is proved (Kanakia et al., 2014; Li et al., 2014; Jasim et al., 2015; Kurantowicz et al., 2015), the GO tendency to persist was found in both human relevant simulated biological fluids (e.g., gastro-intestinal fluids (Guarnieri et al., 2018)) and epithelial *in vitro* models (such as gut and lung (Guarnieri et al., 2018; Di Cristo et al., 2020)). Based on this evidence, the assessment of the potential GO impact on the liver, considered as the central organ dedicated to xenobiotic metabolism and detoxification, is a fundamental step in the innovation process of GO-based technologies. Remarkably, a clear depiction of GO-induced liver toxicity has not been provided yet.

Emergent data on GO hepatotoxicity *in vitro* indicate an IC_50_ approximately within the range from 50 to 100 μg/ml GO (Chatterjee et al., 2014; Liu et al., 2016), along with the induction of apoptosis (Chatterjee et al., 2014; Zheng et al., 2018) and the generation of reactive oxygen species (ROS) (Lammel et al., 2013; Chatterjee et al., 2014; Liu et al., 2016; Zheng et al., 2018).

However, the *in vitro* models currently applied are represented mainly by hepatocellular carcinoma-derived cells that are characterized by low gene expression and poor metabolic activity of drug-metabolizing enzymes (Westerink and Schoonen 2007; Tolosa et al., 2016). It emerges a knowledge lack about the GO impact on specialized hepatic functions (such as phase-I and -II drug-metabolizing enzymes, phase-III efflux transporters, and Albumin), which, among many others, are important markers of drug-induced liver toxicity. For their metabolic and functional features, primary human hepatocytes (PHH) represent the gold standard for studying drug metabolism and toxicity, but their limited availability and technical drawbacks significantly hamper their use at a large scale (Levy et al., 2015; Feng et al., 2019; Tolosa et al., 2019). As an alternative, human HepaRG™ cells, an organotypic co-culture model of hepatocyte- and cholangiocyte-like cells, are reported to exhibit a differentiated phenotype, characterized by a good metabolic activity of drug-metabolizing enzymes (Anthérieu et al., 2012; Nelson et al., 2017). Recently, Strojny et al. showed a clear impairment of the cytochrome P450 (CYP) system in these cells, while no direct cytotoxic effects were evidenced upon treatment with GO or other carbon-based nanostructures (Strojny et al., 2018). In addition, using a microsomal-based model, they corroborated these findings, reporting the inhibition of CYPs metabolic activity by the same nanostructures. A similar impact on CYP isoforms, in terms of gene expression or metabolic activity, was also found for metallic nanoparticles (Au-NPs (Choi et al., 2017), Ag-NPs (Kulthong et al., 2012)), polystyrene nanoparticles (Fröhlich et al., 2010), and single-walled carbon nanotubes (Hitoshi et al., 2012; El-Sayed et al., 2016) even though different experimental approaches were carried out.

The mechanisms underlying the CYP impairment are quite unknown so far. Emerging data indicate that metallic nanoparticles or single-walled carbon-based nanotubes may likely inhibit the metabolic activity of CYPs via direct interaction with enzymes, even though such finding derives from *in silico* simulations or *in vitro* acellular models (e.g., microsomal-based models) (Ye et al., 2014; El-Sayed et al., 2016; Wasukan et al., 2019). Besides, poor information is available on the mechanism occurring *in vivo*. Animal models show that many nanoparticles (such as Ag-NPs, TiO_2_-NPs, Au-NPs, Si-NPs, and multi-walled carbon nanotubes) may affect the cytochrome P450 system at the level of gene expression and metabolic activity (Iavicoli et al., 2016). Rats orally exposed to increasing doses of nano-copper (100, 200, and 400 mg/kg) showed a dose-dependent inhibition of gene expression and metabolic activity of many CYP isoforms (CYP1A2, 2C11, 2D6, 2E1, and 3A1) (Tang et al., 2018). In this study, the highest dose (400 mg/kg) induced oxidative stress and inflammatory cytokine production (IL-2, IL-6, IFN-γ, and MIP-1), which likely determined the concomitant downregulation of pregnane X receptor (PXR), constitutive androstane receptor (CAR), and aryl hydrocarbon receptor (AHR) (Tang et al., 2018), known as key transcription regulators of CYP expression (Evans and Mangelsdorf 2014; Mackowiak and Wang 2016; de Jong et al., 2020). To the best of our knowledge, poor *in vivo* data are available for GO accounting for CYP impairment. Wu et al. found that GO-exposed mice showed a disordered zonation pattern of CYP7A1, 1A1, and 3A7, due to a differential GO localization across hepatic lobules, indicating a detrimental interaction between GO and hepatocytes (Wu et al., 2020). Therefore, it is known that mice exposed to GO by intratracheal instillation displayed acute pulmonary inflammation, pulmonary and hepatic acute-phase response (APR), and genotoxicity (Bengtson et al., 2017). In addition, the transcriptome profiling in GO-exposed mice revealed that, in the lung, the family of acute-phase proteins (APPs) was among the top regulated pathways by GO exposure (Poulsen et al., 2021).

Notably, it has been widely reported using both *in vitro* and *in vivo* models that inflammation strongly impacts the cytochrome P450 system via a multitude of complex pre- and post-transcriptional mechanisms, which depend on specific mediators (for example, IL-6) and target CYP isoforms (de Jong et al., 2020; Stanke-Labesque et al., 2020; Lenoir et al., 2021). In particular, Jover et al. demonstrated that IL-6 is capable of downregulating CYP3A4, providing also the relative molecular mechanism (Jover et al., 2002). Being the major regulator of the hepatic acute-phase response, IL-6 provokes the hepatic secretion of “positive” APPs, such as C-Reactive Protein (CRP), Serum Amyloid Protein A1 (SAA1), complement C3 (c3), Haptoglobin (Hp), and Fibrinogen (Fg), and a concomitant reduced production of “negative” APPs, such as Albumin, Transferrin, and Fibronectin (Castell et al., 1989; Gabay and Kushner 1999). Interestingly, correlating with the inflammation degree, the blood concentrations of APPs are used in clinical practice as biomarkers of inflammation and, in some cases, as indicators of drug-metabolism dysfunctions involving CYP suppression (Stanke-Labesque et al., 2020).

Within this framework, we hypothesize that GO may cause the impairment of the cytochrome P450 system via activation of an acute-phase response with the modulation of positive and/or negative APPs, as it typically occurs and has been demonstrated for IL-6-type cytokines. To corroborate such a hypothesis, in this study, we monitored the GO impact on four representative CYPs using human differentiated hepatocytes generated by upcyte^®^ technology. In parallel, the modulation of two APPs possibly involved in the activation of early acute-phase response (i.e., CRP and Albumin) was quantified both at the level of gene expression and protein production upon GO treatment (IL-6 was used as positive control). Upcyte^®^ hepatocytes employed here are obtained from PHH after transduction with HPV E6 and E7 genes, followed by a positive selection of slowly proliferating OSM-responsive cells (Levy et al., 2015). After OSM removal, they terminally differentiate in 4 days. As a result, they are not immortalized, present a normal karyotype, and do not display a transformed phenotype. Differentiated upcyte^®^ hepatocytes exhibit a mature hepatic phenotype, characterized by sustained catalytic activities of phase-I CYPs and phase-II conjugating enzymes (Schaefer et al., 2016; Tolosa et al., 2016). Notably, CYP3A4 has been shown to have a comparable enzymatic activity (Tolosa et al., 2016) and a similarly robust drug induction (Levy et al., 2015; Tolosa et al., 2016) in upcyte^®^ hepatocytes compared to PHH. Moreover, upcyte^®^ hepatocytes retain key hepatic functions, such as lipid and glycogen accumulation (Tolosa et al., 2016) and Albumin production (Levy et al., 2015; Tolosa et al., 2016) at levels similar to PHH and exhibit correctly a functional epithelial polarization (Levy et al., 2015). Some differences are known between upcyte^®^ hepatocytes and PHH which relate to sinusoidal solute carrier transporters, characterized by lower relative transcript and protein levels compared to PHH, while canalicular efflux pumps has been reported to be well preserved (Schaefer et al., 2018). Nonetheless these limitations, upcyte^®^ hepatocytes have been shown to be a valuable *in vitro* tool for drug-interaction studies (Ramachandran et al., 2015) and for assessing acute and long-term hepatotoxicity (Tolosa et al., 2016; Tolosa et al., 2019). Regarding the progressive loss of specific hepatic features exhibited by PHH after their isolation, phenomenon called dedifferentiation (Heslop et al., 2017; Kiamehr et al., 2019), it has been reported that upcyte^®^ hepatocytes are characterized by a stable or even increased activity of some phase-I (CYP2B6, 2C9, 2E1, and 3A4) and phase-II (UGT1A1 and 2B7) enzymes over time up to 21 days (Donato et al., 2022). Analogously, the Albumin production was almost constant over time (10 days) in differentiated cells (Levy et al., 2015), thus indicating phenotypic stability. Based on these functional and toxicological analogies with PHH and considering their stable hepatic functionality, upcyte^®^ hepatocytes are herein proposed as a suitable hepatic cell model for assessing liver toxicity. In our investigations, 2D cultures of upcyte^®^ hepatocytes were acutely exposed to increasing concentrations of GO in order to evaluate their potential effects on cell viability, apoptosis activation (intrinsic) inflammatory response, oxidative stress induction, and, finally, hepatocyte-specific functions (CYPs gene expression and metabolic activity coupled with APPs modulation). Consequently, our study provides valuable insights into GO hepatotoxicity and related mechanism of action, in terms of stress-related responses and impact on specific hepatic functions, using only one cell model of human differentiated, functional hepatocytes. This broad description of the GO effects is a novel approach for assessing nanomaterial-induced hepatotoxicity.

## Results

### Characterization of Graphene Oxide Suspensions in Complete HHPM

**FIGURE 1:**
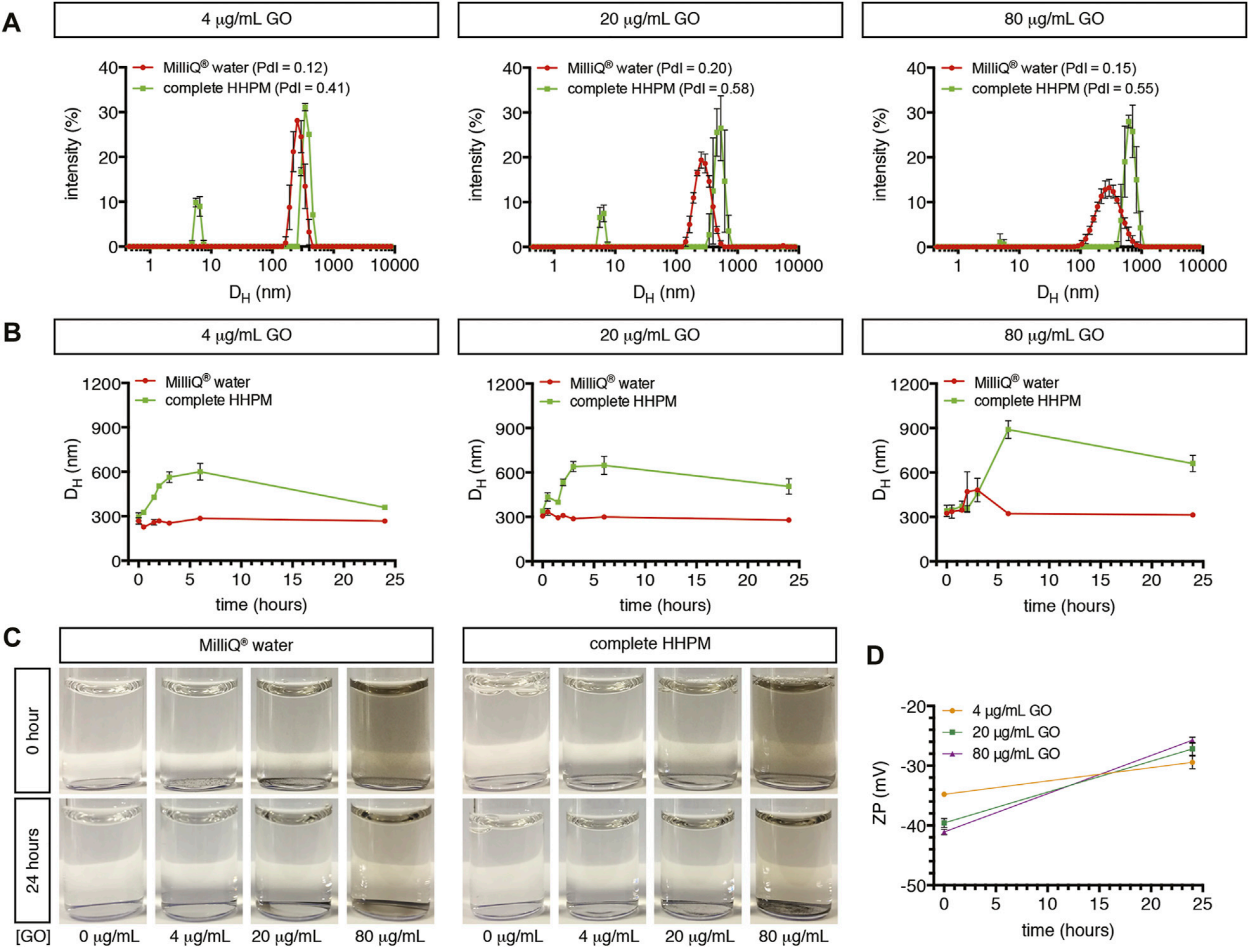
Characterization of GO dispersed in Milli-Q^®^ water or complete HHPM. **(A)** Size distribution profiles by DLS analysis of GO dispersed in Milli-Q^®^ water (red curves) or complete HHPM for 24 h (green curves). Measurements refer to three GO concentrations. **(B)** Temporal stability of the main GO population when dispersed in Milli-Q^®^ water (red curves) or complete HHPM (green curves) at three concentrations over time (up to 24 h) assessed by DLS analysis. **(C)** Representative images of the GO stock suspension diluted in Milli-Q^®^ water and complete HHPM at three GO concentrations after 0 or 24 h of incubation, for visualizing the deposition of sediments on the bottom. **(D)** Surface charge by Zeta Potential analysis of bare GO in Milli-Q^®^ water (at 0 h) and of particle-corona complexes derived from a 24-h incubation of GO in complete HHPM and re-dispersed in Milli-Q^®^ water (as reported by Maiorano et al. (Maiorano et al., 2010)).

GO used in our investigations showed the typical flake-like shape, as shown by SEM and TEM images (Supplementary Figure S1A,C). This shape is in line with previous observations reported by our group on the same material (Guarnieri et al., 2018; Di Cristo et al., 2020). The elemental analysis by EDS revealed a high content of oxygen atoms in the carbon-based structure of GO (Supplementary Figure S1B), confirming the graphene oxidation, as also shown by the previously reported thermogravimetric analysis of GO (Guarnieri et al., 2018; Di Cristo et al., 2020). Colloidal stability of GO suspensions in cell stimulation-like conditions was assessed using complete HHPM as dispersant medium at three representative GO concentrations (4, 20, 80 μg/ml). After a 24-h incubation (corresponding to an acute stimulation of upcyte^®^ hepatocytes), 4 μg/ml GO suspension presented two main peaks with a relative mean size of 6.0 ± 0.2 nm (peak #1) and 359.5 ± 1.3 nm (peak #2; Figure 1A), respectively. The first is identified by and assigned to the protein components supplemented in complete HHPM (Maiorano et al., 2010; Magrì et al., 2018) (Supplementary Figure S1D). Peak #2 corresponds, therefore, to the main GO population, with a relative intensity equal to 80.0%. The peak identity is further confirmed by the presence of a peak at a similar position shown by the GO sample dispersed in Milli-Q^®^ water at the same concentration even though a smaller relative mean size was evident (267.4 ± 15.8 nm; Figure 1A). At the same incubation time, as GO concentration increases (20 and 80 μg/ml), it is noticed a shift of peak #2 toward larger sizes in complete HHPM (505.6 ± 51.7 nm and 660.9 ± 55.5 nm, respectively; Figure 1A), along with a progressive rise of the relative peak intensity (85.4 and 98.3%, respectively). In Milli-Q^®^ water, the corresponding peaks were slightly stable, alongside the relative peak intensities. Peak position and relative intensity of all peaks identified in each tested condition are reported in Supplementary Table S1. Furthermore, consecutive DLS measurements (0–24 h) were acquired to follow the possible changes of the relative mean size of peak-#2 GO, likely due to suspension instability. Results indicate that, especially for the highest concentration tested, 80 μg/ml, the mean size rapidly increased in the first 6 h in complete HHPM, leaving a slight decrease at 24 h (Figure 1B). This effect is not observed when GO was incubated in Milli-Q^®^ water, where the mean size remained almost stable within the 24 h. Overall, these data, along with the occasional emergence of a peak #3 at the highest concentrations (20–80 μg/ml) characterized by relative mean sizes of 4.0–5.0 µm (see Supplementary Table S1), suggest that GO suspensions especially in complete HHPM are poly-dispersed and show a tendency to agglomerate/aggregate within the temporal range studied (0–24 h). The agglomeration/aggregation tendency of GO has been further evidenced by the presence of sediments at the highest concentrations (20–80 μg/ml), as reported in Figure 1C. The zeta potential analysis revealed a negative surface charge of GO in Milli-Q^®^ water, which goes from -35 mV to -41 mV as a function of the concentration applied (Figure 1D). After 24-h incubation in complete HHPM, the surface charge of GO increased to about −27.5 mV, as a consequence of the possible formation of a bio-macromolecular corona around GO (Maiorano et al., 2010). The particle-corona complexes (see technical details in Materials and Methods) show mean hydrodynamic diameters almost comparable to bare GO (Supplementary Figure S1E).

### Graphene Oxide Impacts Cell Viability and Induces Membrane Damage in Upcyte^®^ Hepatocytes

**FIGURE 2:**
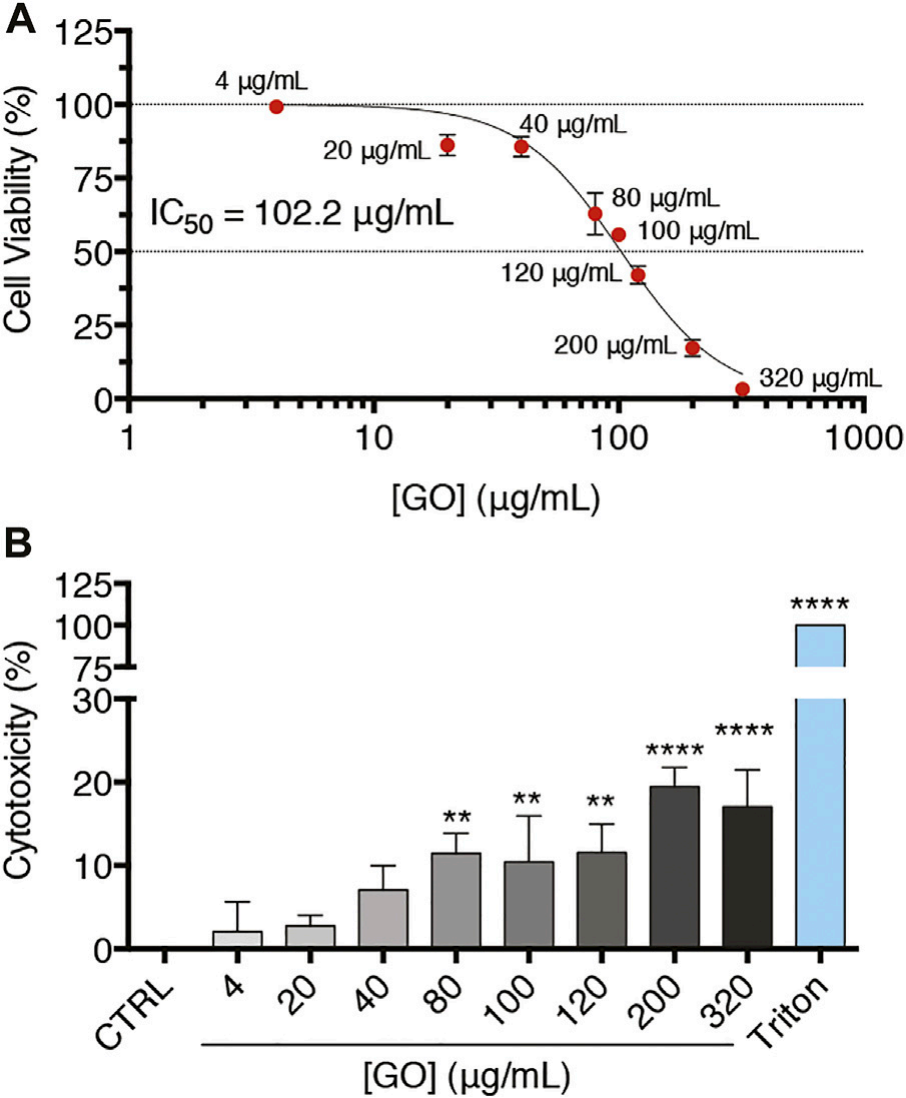
Confluent upcyte^®^ hepatocyte cultures treated with increasing GO concentrations for 24 h. **(A)** Cell viability of GO-treated cells after 24 h of incubation evaluated by resazurin reduction assay. Results are expressed as percentage values compared to the control conditions (indicated as CTRL). Results represent means ± SD of three independent experiments. The half maximal inhibitory concentration (IC_50_) of GO was calculated using the log (inhibitor) vs. normalized response curves model (R^2^ = 0.98). **(B)** Cell membrane damage by cytotoxicity assay in GO-treated cells after 24 h of incubation. “Triton” refers to the positive control, i.e., cells treated with 0.03% Triton X-100 for 24 h, while “CTRL” represents untreated cells. Results are expressed as percentage values compared to Triton (set as 100%) after having removed the basal value of CTRL (set as 0%). Results were obtained from three independent experiments (means ± SD). The symbols ‘**’ and “****” refer to *p* ≤ 0.0038 and *p* < 0.0001, respectively, calculated versus CTRL (ordinary one-way ANOVA).

To understand the possible detrimental impact of GO on upcyte^®^ hepatocytes, we first evaluated its impact on cell viability and cell membrane integrity. Confluent cell cultures were treated for 24 h with increasing GO concentrations, as reported in Figure 2A. The half-maximal inhibitory concentration (IC_50_) was calculated testing a wide concentration range of GO (4–320 μg/ml). As evidenced by the reduction of the metabolic activity, GO determined a dose-dependent decrease of cell viability, with an IC_50_ equal to 102.2 μg/ml (Figure 2A). Note that the highest GO concentration tested, corresponding to 320 μg/ml, caused a cell viability reduction comparable to the lethal effect exerted by the positive control, which was 0.03% Triton X-100 (Supplementary Figure S2A). As revealed by the cytotoxicity assay, treated cells also showed a significant release in the culture medium of the cytosolic lactate dehydrogenase (LDH), indicating cell membrane damage (Figure 2B). In particular, at 80 μg/ml GO, a concentration value lower than the IC_50_, it is possible observing a significant increase of cytotoxicity to about 11.5% (*p* = 0.0015) with respect to the control, whereas at lethal doses (320 μg/ml), the damage weakly increased only up to 17% (*p* < 0.0001). In addition, with the increase of GO concentration, the decrease of cell viability alongside the rise in released LDH showed a significant negative linear correlation (*p* < 0.0001 and Pearson’s *r* = 0.9521; Supplementary Figure S2B). As a function of the GO concentration, the progressive deposition of GO on the top surface of treated cells was observed. In fact, the number of GO deposits and the percentage area covered by GO dose-dependently increased (Supplementary Figures S2C–E). Considering three representative GO concentrations (4, 20, and 80 μg/ml), the mean lateral size relative to GO deposits by SEM images analysis was about 2.0 μm, with no differences among the GO concentrations (Supplementary Figures S2F,G). Nevertheless, the presence of large deposits does not likely prevent the uptake of the nano-sized GO present in suspension. The GO deposition is indicative of a partial colloidal instability of GO suspensions (as also evidenced by DLS analysis), so mechanical cell damage due to particle deposition cannot be excluded.

### Graphene Oxide Induces Early Apoptosis but Not Oxidative Stress in Upcyte^®^ Hepatocytes at Sub-IC_50_ Doses

**FIGURE 3:**
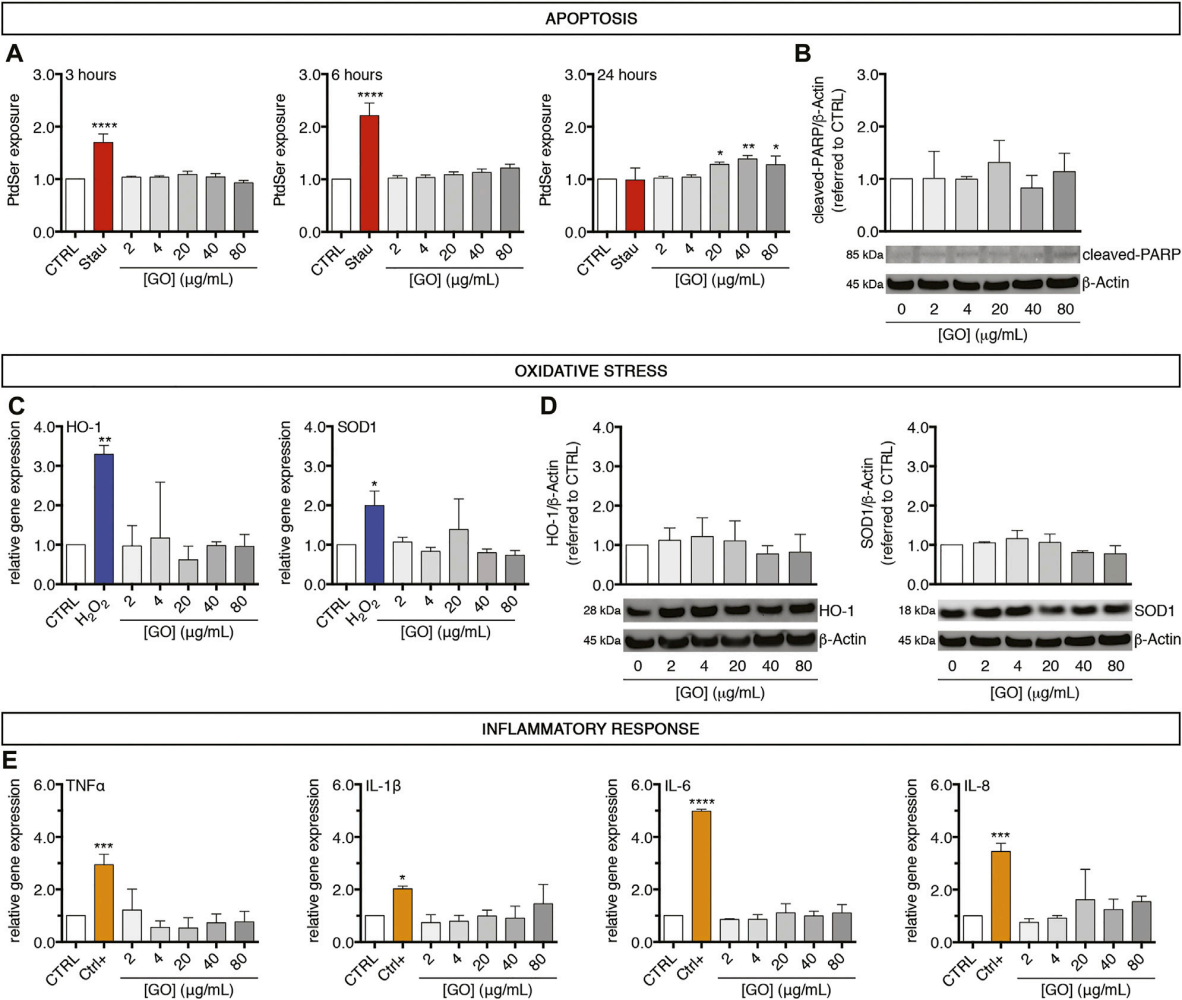
A 24-h exposure of GO induces early weak apoptosis but not oxidative stress nor inflammation in upcyte^®^ hepatocytes treated at sub-lethal concentrations. **(A)** Temporal exposure of PtdSer in GO-treated cells evaluated at different time points (3, 6, and 24 h) by apoptosis assay. “Stau” refers to the positive control, represented by cells treated with 0.5 μM Staurosporine for 24 h. Results are expressed as an n-fold increase over the control condition (indicated as CTRL) for each incubation time. Results represent means ± SD of three independent experiments. The symbols “*”, “**” and “****” refer to *p* ≤ 0.0429, *p* = 0.0043 and *p* < 0.0001, respectively, calculated versus CTRL (ordinary one-way ANOVA). **(B)** Protein levels of cleaved-PARP in GO-treated cells in comparison to CTRL, assessed by western blot. Densitometric analysis of three blots derived from independent experiments (means ± SD; upper panel) and a representative blot (lower panels) are reported. *β*-Actin was used as the internal control to normalize densitometric values of all culture conditions. **(C)** Relative gene expression of cellular antioxidants HO-1 (left) and SOD1 (right) in GO-treated cells versus CTRL, analysed by qPCR. “H_2_O_2_” refers to the positive control, represented by cells treated with 400 μM H_2_O_2_ overnight. Results are expressed as means ± SD values of three independent experiments. The symbols ‘*’ and ‘**’ refer to *p* = 0.0269 and *p* = 0.0094, respectively, calculated versus CTRL (ordinary one-way ANOVA). **(D)** Protein levels of HO-1 (left) and SOD1 (right) in GO-treated cells compared to CTRL, assessed by western blot. For each marker, densitometric analysis of three blots derived from independent experiments (means ± SD; upper panel) and a representative blot (lower panels) are reported. *β*-Actin was used as the internal control to normalize densitometric values of all culture conditions. **(E)** Relative gene expression of four inflammation mediators (TNFα, IL-1β, IL-6, and IL-8) in GO-treated cells versus CTRL, analysed by qPCR. “Ctrl+” refers to the positive control (see technical details in Materials and Methods). Results are expressed as means ± SD values of three independent experiments. The symbols “*”, “***” and “****” refer to *p* = 0.0482, *p* ≤ 0.0008 and *p* < 0.0001 respectively, calculated versus CTRL (ordinary one-way ANOVA).

To assess apoptosis, oxidative stress, or inflammatory response potentially triggered by GO, upcyte^®^ hepatocytes were treated with increasing sub-IC_50_ doses (2–80 μg/ml). Regarding apoptosis induction, treated cells were screened to follow the extent of phosphatidylserine (PtdSer) exposure over time as early indicator of apoptosis (Figure 3A), finding no significant variations at any of the tested concentrations for the early GO treatment times (3 and 6 h). After 24 h of treatment, however, PtdSer exposure significantly increased about 1.3-fold with respect to the control upon the highest concentrations (20 μg/ml GO, *p* = 0.0352; 40 μg/ml GO, *p* = 0.0043; and 80 μg/ml GO, *p* = 0.0429). It is important to underline that GO showed just mild induction of PtdSer exposure and a different kinetic compared to the positive control (0.5 μM Staurosporine). Indeed, Staurosporine induced an earlier significant increase of PtdSer exposure, reaching a maximum peak at 6 h (*p* < 0.0001). PtdSer level reached the basal value again after 24 h of Staurosporine treatment in correspondence to the significant increase of necrotic cells (*p* < 0.0001, Supplementary Figure S3). For evidencing a further sign of cellular apoptosis, the intracellular amount of cleaved-PARP in GO-treated cells was measured. We found no alteration of cleaved-PARP levels with respect to the control at any of the tested concentrations, further confirming the mild apoptotic effect induced by GO (Figure 3B). Since oxidative stress is involved in many mechanisms of cytotoxicity (such as apoptosis, DNA damage, lipid peroxidation), we investigated the possible role of GO as an oxidative stress inducer (Di Cristo et al., 2018). We found that GO did not modulate the gene expression of two main cellular antioxidants, HO-1 and SOD1, at any of the tested concentrations (Figure 3C). By contrast, the positive control (400 μM H_2_O_2_) induced a significant upregulation of HO-1 and SOD1 gene expression (*p* = 0.0094 and *p* = 0.0269, respectively). Upon GO treatment, western blot analysis of HO-1 and SOD1 confirmed the absence of significant variations of the corresponding proteins with respect to the control (Figure 3D). As far as an inflammatory response is concerned, GO did not induce in our cell model any modulation of the gene expression of TNFα, IL-1β, IL-6, and IL-8, considered mediators of inflammation (Figure 3E). On the other hand, upcyte^®^ hepatocytes showed a significant upregulation of all those genes (*p* = 0.0008 for TNFα; *p* = 0.0482 for IL-1β; *p* < 0.0001 for IL-6; *p* = 0.0001 for IL-8), when exposed to exogenous pro-inflammatory stimuli (see technical details in Materials and Methods). These data indicate that the cell model employed has a poor ability for inflammatory response; however, based on only this evidence, we cannot conclude that GO does not induce inflammation *in vivo*.

### Graphene Oxide Impairs Cytochrome P450 System in Upcyte^®^ Hepatocytes at Sub-lethal Concentrations

**FIGURE 4:**
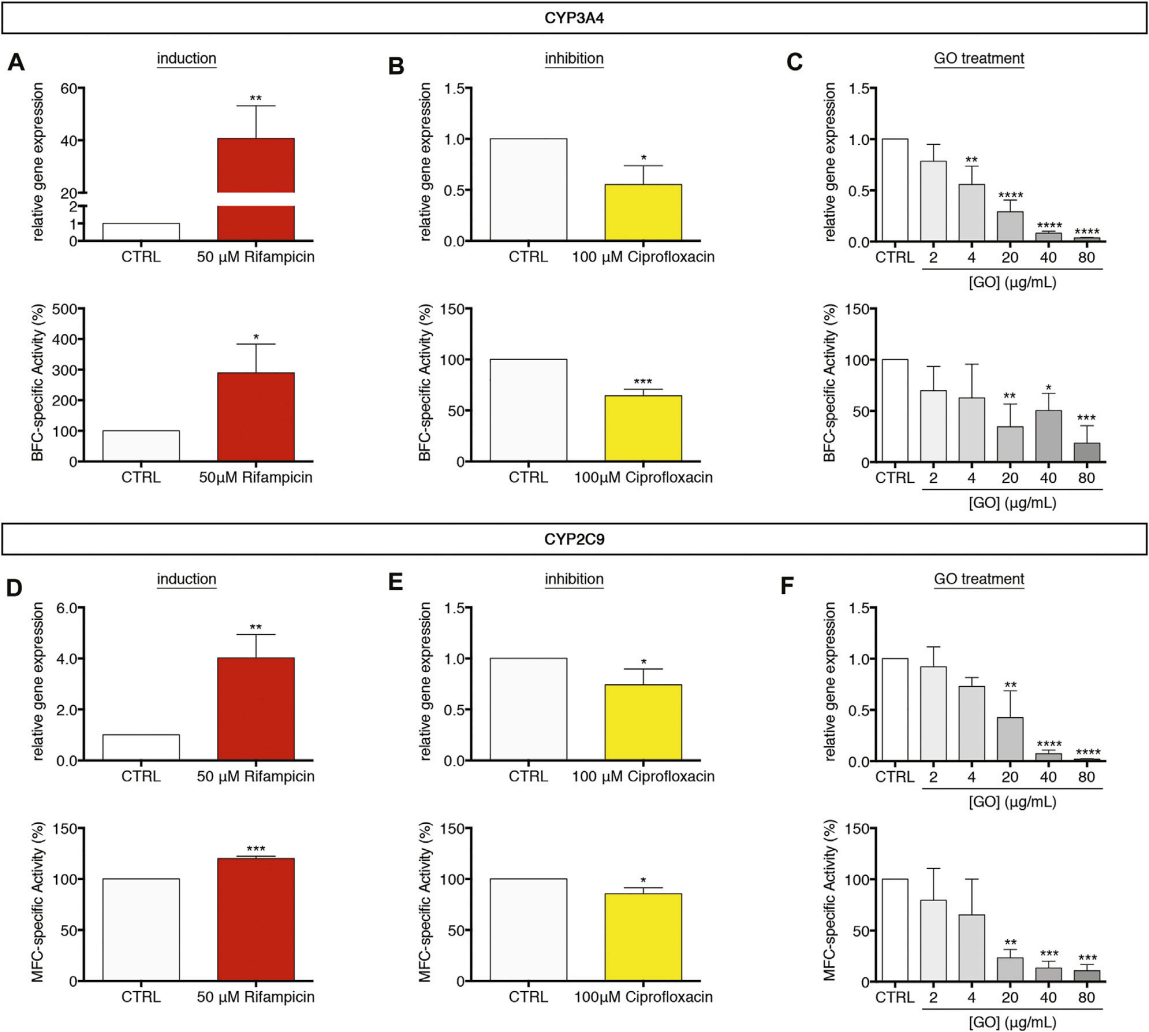
Modulation of the cytochrome P450 system in upcyte^®^ hepatocytes upon sub-lethal GO exposure. **(A,B)** Relative gene expression and BFC-specific metabolic activity of CYP3A4 in cells treated daily up to 72 h with 50 μM Rifampicin **(A)** or 100 μM Ciprofloxacin **(B)** in comparison to untreated cells (indicated as CTRL). Results are expressed as means ± SD values of three independent experiments. The symbols “*”, “**” and “***” refer to *p* ≤ 0.0247, *p* = 0.0052 and *p* = 0.0006, respectively, calculated versus CTRL (unpaired *t*-Test). **(C)** Relative gene expression and BFC-specific metabolic activity of CYP3A4 in GO-treated cells in comparison to CTRL. Results are expressed as means ± SD values of three independent experiments. The symbols “*”, “**”, “***” and “****” refer to *p* = 0.0164, *p* ≤ 0.0017, *p* = 0.0002 and *p* < 0.0001 respectively, calculated versus CTRL (ordinary one-way ANOVA). **(D,E)** Relative gene expression and MFC-specific metabolic activity of CYP2C9 in cells treated daily up to 72 h with 50 μM Rifampicin **(D)** or 100 μM Ciprofloxacin **(E)** in comparison to CTRL. Results are expressed as means ± SD values of three independent experiments. The symbols“*”, “**” and “***” refer to *p* ≤ 0.0444, *p* = 0.0047 and *p* = 0.0001, respectively, calculated versus CTRL (unpaired *t*-Test). **(F)** Relative gene expression and MFC-specific metabolic activity of CYP2C9 in GO-treated cells in comparison to CTRL. Results are expressed as means ± SD values of three independent experiments. The symbols “**”, “***” and “****” refer to *p* ≤ 0.0019, *p* ≤ 0.0007, and *p* < 0.0001, respectively, calculated versus CTRL (ordinary one-way ANOVA).

For evaluating the GO impact on the cytochrome P450 system of upcyte^®^ hepatocytes, cells were treated for 24 h with increasing sub-IC_50_ GO doses (2–80 μg/ml). We measured the gene expression and the corresponding metabolic activity of CYP3A4 and CYP2C9, which are the most representative enzymes of that system. In parallel, the responsiveness of cytochromes P450 was assessed, making use of well-known drugs. For this aim, cells were daily incubated up to 72 h (Bjornsson et al., 2003; Fahmi et al., 2008) with 50 μM Rifampicin or 100 μM Ciprofloxacin, which act as inducer or inhibitor of both CYP3A4 and CYP2C9, respectively. Results show that, upon treatment with Rifampicin, CYP3A4 gene expression was about 41-fold upregulated with respect to untreated cells (*p* = 0.0052; Figure 4A, upper panel), whereas, when cells were treated with Ciprofloxacin, 0.6-fold downregulated in comparison to the control level (*p* = 0.0138; Figure 4B, upper panel). Similarly, CYP2C9 gene expression was significantly regulated upon the same treatments, with a Rifampicin-mediated upregulation of about 4.0-fold (*p* = 0.0047; Figure 4D, upper panel) and a Ciprofloxacin-mediated downregulation to about 0.7-fold with respect to untreated cells (*p* = 0.0444; Figure 4E, upper panel). Therefore, we evaluated the metabolic activity of CYP3A4 and CYP2C9 using as substrates two fluorescent compounds, 7-benzyloxy-4-trifluoromethylcoumarin (BFC) and 7-methoxy-4-trifluoromethylcoumarin (MFC), respectively. Results indicate that, in line with the gene expression data, the activity of CYP3A4 was statistically increased by about 290% in Rifampicin-treated cells with respect to the control (*p* = 0.0247; Figure 4A, lower panel), whereas it appeared to be reduced to about 64% in the case of Ciprofloxacin-treated cells (*p* = 0.0006; Figure 4B, lower panel). Comparably, CYP2C9 activity was modulated by the same treatments consistently with gene expression regulation (Figures 4D,E, lower panels). Afterward, we evaluated the effect of sub-IC_50_ concentrations of GO on the cytochrome P450 system. We observed a dose-dependent downregulation of CYP3A4 gene expression (starting from 4 μg/ml, *p* = 0.0015), which was equal to about 0.37-fold with respect to the control level at the highest concentration (80 μg/ml GO, *p* < 0.0001; Figure 4C, upper panel). Consistent with the gene expression, CYP3A4 metabolic activity showed a significant and progressive dose-dependent reduction, reaching a level of about 18.6% with respect to the control, at a concentration value of 80 μg/ml GO (*p* = 0.0002; Figure 4C, lower panel). In parallel, CYP2C9 gene expression significantly decreased starting from 20 μg/ml GO (*p* = 0.0012), down to about 1.9% with respect to the control level with 80 μg/ml GO (*p* < 0.0001; Figure 4F, upper panel). CYP2C9 metabolic activity was also significantly reduced, reaching about 10.9% of the basal level with the highest GO concentration (80 μg/ml, *p* = 0.0006; Figure 4F, lower panel). For both CYP3A4 and CYP2C9, GO-mediated gene expression downregulation had a significant positive linear correlation with the metabolic activity impairment (*p* = 0.0119 and Pearson’s *r* = 0.9095 for CYP3A4 and *p* = 0.0015 and Pearson’s *r* = 0.9682 for CYP2C9; Supplementary Figure S4A, B). Our results indicate a strong inhibition activity of GO on CYP3A4 and CYP2C9 gene expression and relative metabolic activities. Such an inhibitory response appears to be dose-dependent, and the negative effect of 4 μg/ml GO could be approximately compared to that induced by 100 μM Ciprofloxacin (∼33 μg/ml). The inhibition of the cytochrome P450 system by sub-lethal doses of GO was also confirmed on CYP2B6 and CYP1A2 (Supplementary Figures S4E, H). For CYP2B6, gene expression decreased starting from 20 μg/ml GO (*p* = 0.0069), while GO affected CYP1A2 gene expression at lower doses (2 μg/ml; *p* = 0.0052). Upon 80 μg/ml GO treatment, CYP2B6 and CYP1A2 reached a gene expression equal to about 4.3 and 11.3% of the control level (*p* < 0.0001 for both), respectively. In contrast to the effects exerted on CYP3A4 and CYP2C9 gene expression, Rifampicin and Ciprofloxacin had no effects on CYP2B6 and CYP1A2, revealing their specific mechanism of action only on CYP3A4 and CYP2C9 in upcyte^®^ hepatocytes (Supplementary Figures S4C, D and Supplementary Figures S4F, G). Conversely, GO seems to act on a wide range of targets within the cytochrome P450 system.

### Graphene Oxide Does Not Modulate Gene Expression of Phase-II GST and Phase-III ABCG2

**FIGURE 5:**
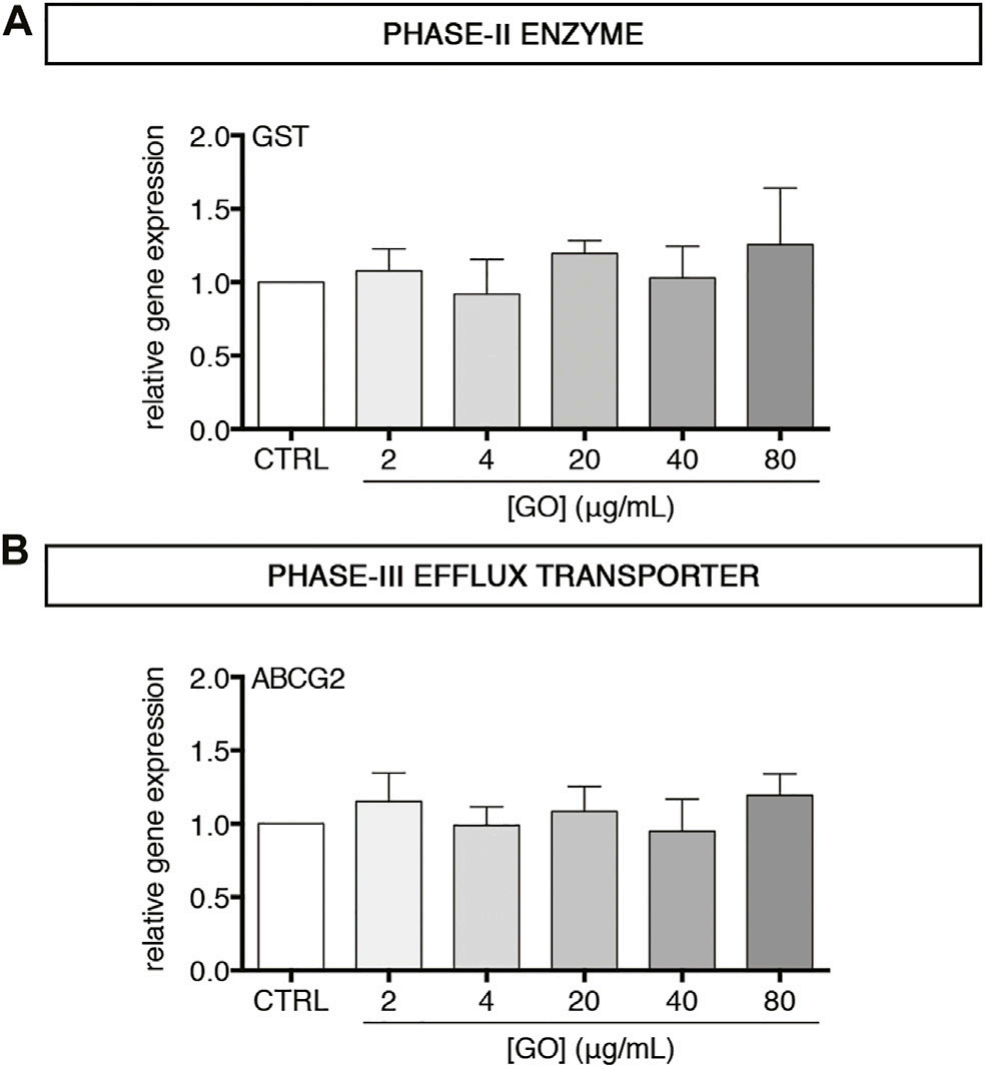
Effect of sub-lethal GO doses on transcript levels of two representative executioners of phase–II and–III drug/xenobiotic metabolism in upcyte^®^ hepatocytes treated for 24 h **(A–B)** Relative gene expression of GST **(A)** and ABCG2 **(B)** in GO-treated cells versus untreated cells (indicated as CTRL). Results are expressed as means ± SD values of three independent experiments.

After having observed the effects of GO on phase-I drug metabolism enzymes, we investigated the impact of sub-lethal GO exposures on phase-II and phase-III drug metabolism/transport enzymes in upcyte^®^ hepatocytes. In particular, the gene expression of Glutathione S-Transferase (GST) and ATP Binding Cassette Subfamily G Member 2 (ABCG2) was evaluated. Results show that GST and ABCG2 gene expression was not modulated by GO at any of the tested concentrations (Figures 5A,B). For confirming that GO effects observed on phase-I CYPs were specific and not related to a global de-differentiation of upcyte^®^ hepatocytes, we also evaluated in control and GO-treated cells the expression of Glucose Transporter 2 (GLUT2), which is a solute carrier transporter catalysing the passive transport of glucose across the cell membrane in functional hepatocytes (Kim et al., 1998; Thorens 2015). FACS analysis revealed a stable expression of GLUT2 over time in the control cells, confirming the phenotypic stability of the cell model employed, and only a moderate or absent modulation by GO, depending on the dose (Supplementary Figure S5A). Regarding the xenobiotic-sensing receptors, PXR and CAR, which are transcription regulators of a large part of phase-I, -II, and -III executioners (Evans and Mangelsdorf 2014), we observed that PXR was significantly downregulated only by 80 μg/ml GO (*p* = 0.0224), while CAR gene expression was not modulated at the experimental doses applied in this study (Supplementary Figures S5B, C).

### Graphene Oxide Downregulates Gene Expression of Albumin and Reduces the Corresponding Intracellular Protein Amount

**FIGURE 6:**
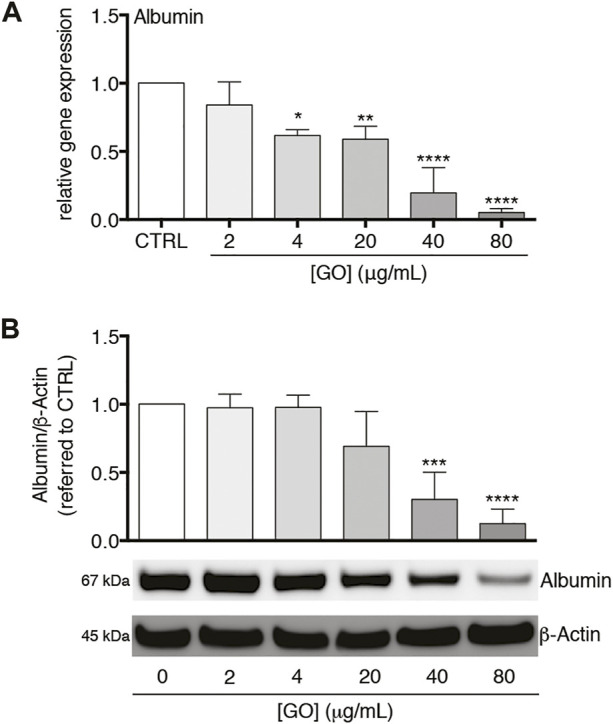
Effects of sub-lethal GO doses on Albumin gene expression and translation in upcyte^®^ hepatocytes. **(A)** Relative gene expression of Albumin in GO-treated cells versus untreated cells (indicated as CTRL). Results are expressed as means ± SD values of three independent experiments. The symbols“*”, “**” and “****” refer to *p* = 0.0155, *p* = 0.0046 and *p* < 0.0001, respectively, calculated versus CTRL (ordinary one-way ANOVA). **(B)** Intracellular Albumin level in GO-treated cells in comparison to CTRL, assessed by western blot. Densitometric analysis of three blots derived from independent experiments (means ± SD; upper panel) and a representative blot (lower panels) are reported. *β*-Actin was used as the internal control to normalize densitometric values of all culture conditions. The symbols “***” and “****” refer to *p* = 0.0004 and *p* < 0.0001, respectively, calculated versus CTRL (ordinary one-way ANOVA).

We also analysed the impact of sub-lethal GO doses on Albumin gene expression and translation. In the case of gene expression, a dose-dependent downregulation was observed with a resulting reduction to about 5.2% with respect to the control transcript level by 80 μg/ml GO (*p* < 0.0001; Figure 6A). A similar trend in the Albumin transcript translation was found, with a reduction of the intracellular protein amount equal to about 12.5% of the control by the highest GO dose (*p* < 0.0001; Figure 6B). Considering two other hepatic proteins, GO had a dose-dependent negative impact on the gene expression of Transferrin and Transthyretin with a downregulation equal to about 16.5 and 16.4% of the control, respectively, by 80 μg/ml GO (*p* = 0.0051 and *p* = 0.0023, respectively; Supplementary Figure S6A, B).

### Graphene Oxide Induces the Gene Expression and the Secretion of C-Reactive Protein at Early Times

Having in mind that inflammation and its mediators *in vivo* determine the inhibition of cytochromes P450 via a multitude of complex pre- and post-transcriptional mechanisms (de Jong et al., 2020; Stanke-Labesque et al., 2020; Lenoir et al., 2021), and considering that at the experimental conditions applied (i.e., sub-lethal doses) GO did not stimulate an inflammatory response in our cell model (see above), we assessed if GO may display a pro-inflammatory cytokine-like behaviour, provoking the impairment of the cytochrome P450 system and, at the same time, the secretion of a “positive” acute-phase protein. In this regard, it has been widely reported that IL-6 acts as the chief regulator of APPs in human hepatocytes during inflammation (Castell et al., 1989), as well as a suppressor of CYP3A4 (Jover et al., 2002). Hence, IL-6 was applied herein as a positive control.

To address the point and verify this hypothesis, upcyte^®^ hepatocytes were treated with a very low, sub-lethal dose of GO (4 μg/ml) in order to minimize as much as possible other toxic effects (such as cell membrane damage and early apoptosis), and the gene expression of CYP3A4 and CRP (Gabay and Kushner 1999) was monitored over time.

**FIGURE 7:**
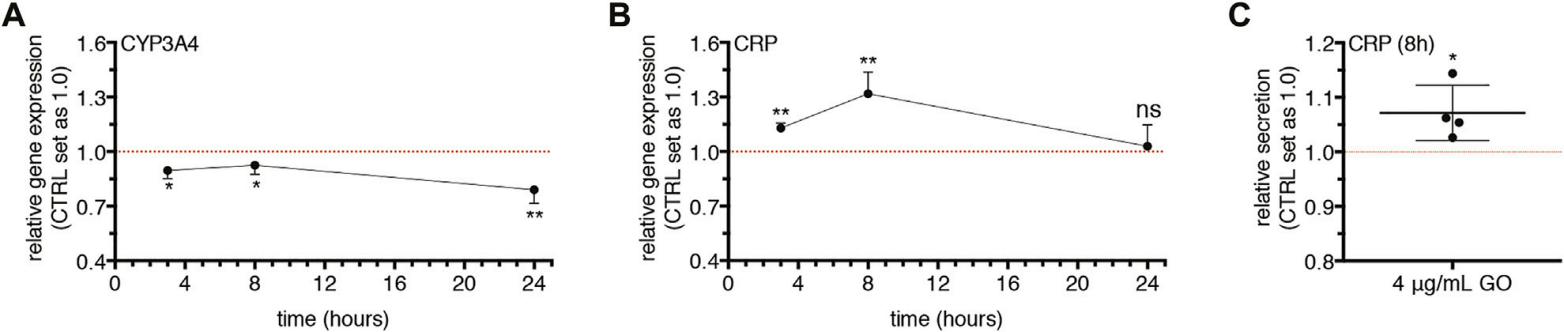
Effects of 4 μg/ml GO on CYP3A4 and CRP gene expression and CRP secretion over time. **(A–B)** Relative gene expression of CYP3A4 **(A)** and CRP **(B)** in GO-treated cells versus untreated cells (indicated as CTRL) at 3, 8, and 24 h of treatment. Results are expressed as means ± SD values of at least three independent experiments. The symbols “*”, “**” and “ns” refer to *p* ≤ 0.0256, *p* ≤ 0.0086 and *p* > 0.05, respectively, calculated versus CTRL (unpaired *t*-Test). **(C)** Relative secretion of CRP in GO-treated cells versus untreated cells (indicated as CTRL) after 8 h of treatment (indicated as 8 h). Results are expressed as mean ± SD value of four independent experiments. The symbol ‘*’ refers to *p* = 0.0303, calculated versus CTRL (unpaired *t*-Test).

We found an early significant downregulation of CYP3A4 after 3 h of treatment (*p* = 0.0165; Figure 7A). The transcript level of CYP3A4 remained comparably reduced at 8 h (*p* = 0.0256) and decreased further at 24 h (*p* = 0.0086). In parallel, the gene expression of CRP was upregulated starting from 3 h (*p* = 0.0012) and reached a maximal peak at 8 h (*p* = 0.0018; Figure 7B). At the end of GO treatment (24 h), the CRP transcript level returned to the basal level. At the peak of CRP upregulation, we found a significantly increased secretion of CRP (*p* = 0.0303; Figure 7C). Similarly, after 8 h of treatment, 20 ng/ml rhIL-6 serving as positive control provoked the downregulation of CYP3A4 (*p* = 0.0006) and the upregulation of CRP (*p* = 0.0057; Supplementary Figure S7A). Accordingly, the secretion of CRP was increased by rhIL-6 at 8 h (*p* = 0.0101; Supplementary Figure S7B).

## Discussion

This study aimed to investigate *in vitro* hepatotoxicity of GO after 24-h acute exposure in a 2D cell-culture model: the second-generation upcyte^®^ hepatocytes. These cells, generated from primary human hepatocytes (PHH) via genetic engineering (Levy et al., 2015), represent an interesting hepatic cell model since they offer the advantages of hepatoma cell lines (easy handling and high availability) and, when differentiated, show a mature hepatic phenotype. After transduction of PHH with HPV E6 and E7 genes, slowly proliferating cells derived from E6/E7^low^ cell colonies are positively selected and expanded upon treatment with OSM. The removal of OSM induces in 4 days terminal differentiation into metabolically functional, polarized hepatocytes similar to PHH (Levy et al., 2015). Hence, they are not immortalised but have a normal karyotype. In addition, upcyte^®^ hepatocytes are suitable for CYPs induction or inhibition studies (Ramachandran et al., 2015) and acute or long-term hepatotoxicity testing (Tolosa et al., 2016; Tolosa et al., 2019). Overall, based on these features, the upcyte^®^ hepatocytes may reproduce the functionalities of primary hepatocytes and can then be considered a reliable *in vitro* cell culture model for studying the acute effects of toxicants on the hepatic tissue. Given the wide range of potential technological applications (including nanomedicine), GO has raised a significant commercial interest. However, the understanding of GO impact on human health is still largely unknown, but it is a fundamental requirement for the safe development of GO-based technologies (Park et al., 2017; Fadeel et al., 2018). Herein, we focused our attention on the potential impact of GO on the hepatic tissue that has a central role in the biotransformation and detoxification of xenobiotics after their entrance into the circulatory system. To address this aim, we selected second-generation upcyte^®^ hepatocytes as hepatic cell model and, specifically, hepatocytes derived from the specific donor 653-03, which, beyond the good basal activity of cytochromes P450 (CYPs), also show appropriate responsiveness to known inducers and inhibitors of CYP enzymes (Ramachandran et al., 2015).

Before assessing *in vitro* the GO toxicity, an extensive physical-chemical characterization of GO was provided in terms of morphology, dimensions, and surface charge using a range of techniques such as SEM/TEM, DLS, and Zeta Potential. When dispersed in Milli-Q^®^ water, GO showed the typical flake-like structure of graphene with a size distribution of about 300 nm and a surface charge ranging from −35 mV to −41 mV, which are comparable to previously published data (Guarnieri et al., 2018; Di Cristo et al., 2020). In complete HHPM, GO adsorbed medium proteins (Maiorano et al., 2010) and had an appreciable tendency for sedimentation.

After that, upcyte^®^ hepatocytes were acutely exposed for 24 h to increasing GO concentrations (4–320 μg/ml). By applying such a wide concentration range, an IC_50_ equal to 102.2 μg/ml was derived, and, at concentrations higher than 80 μg/ml, cell membrane damage was evidenced. These data are in line with a recent study employing the HepaRG™ cell line, which, although with some differences, presents a hepatocyte-like differentiated phenotype (Anthérieu et al., 2012; Nelson et al., 2017). These cells, indeed, showed the absence of cytotoxicity at the concentration of 50 μg/ml (Strojny et al., 2018). To the best of our knowledge, this is the only work that investigated the potential toxicological effect of GO using a cell line presenting features comparable to human hepatocytes, even though HepaRG™ cell line is an organotypic co-culture of hepatocyte- and cholangiocyte-like cells in contrast to upcyte^®^ hepatocytes. Overall, our results indicated a GO-mediated toxicity at a concentration range also comparable to established cell lines. HepG2 cells, when treated with GO, presented an IC_50_ approximately within the range from 50 to 100 μg/ml, along with a clear cell viability reduction at concentration values higher than 80 μg/ml (Chatterjee et al., 2014; Liu et al., 2016). In L02 cells, GO provoked only a moderate cell viability reduction at 100 μg/ml or higher concentrations (Zheng et al., 2018). Taken together, these data indicate that GO-induced toxicological responses in hepatic cells seem to be not particularly affected by cell-type intrinsic features.

Afterward, we studied stress-related cell responses (apoptosis, oxidative stress, and inflammation) in upcyte^®^ hepatocytes upon acute treatment with increasing sub-IC_50_ GO doses (2–80 μg/ml). Upon such a concentration range, we found an increased temporal exposure of PtdSer, but unvaried protein levels of cleaved-PARP. PtdSer is present in the inner layer of the plasma membrane, but it is exposed to the outer layer as an “eat me” signal for macrophages during apoptosis (Segawa and Nagata 2015). PARP is involved in DNA repair in normal conditions, but when it is proteolytically cleaved by the executioner Caspase-3, the apoptotic process is irreversible (Nicholson et al., 1995; Tewari et al., 1995). Based on these molecular mechanisms, the obtained results indicate an early-stage GO induction of apoptosis, which does not undergo maturation (no cell death in upcyte^®^ hepatocytes at the end of the treatment, according to no changes in cleaved-PARP levels). Similarly, it has been reported a dose-dependent increase of early apoptotic cells after 24 h of GO treatment, both in L02 cells (Zheng et al., 2018) and in HepG2 cells (Chatterjee et al., 2014), even though higher GO doses were used in L02 cells (100 and 300 μg/ml) with respect to HepG2 cells (up to 50 μg/ml). Moreover, Chatterjee et al. reported that apoptosis executioners (Caspase-8, -9, and -3) were unaffected or even downregulated after 24 h of treatment with both 20 μg/ml and 81 μg/ml of GO, showing that late apoptotic cells increased in number only after longer treatments (48 h) (Chatterjee et al., 2014). Concerning intracellular antioxidant defences in upcyte^®^ hepatocytes, we found no modulation of both gene expression and protein levels of cellular antioxidants HO-1 and SOD1 after 24 h of GO treatment, suggesting that GO should have induced no or weakly the generation of endogenous reactive oxygen species (ROS). However, some recent studies show ROS generation after different times of exposure in both HepG2 cells and L02 cells, with differences in treatments and collection times (Lammel et al., 2013; Chatterjee et al., 2014; Liu et al., 2016; Zheng et al., 2018). Among these studies, only Chatterjee et al. investigated also the effect of GO on the antioxidant defences of HepG2 cells, showing GO-induced repression of SOD1, CAT, and GSTA1 gene expression (Chatterjee et al., 2014). In this respect, upcyte^®^ hepatocytes could be less susceptible to GO-induced oxidative stress than established hepatic cells, or their antioxidant defenses could be sufficient to neutralize a mild generation of ROS. Finally, we evaluated the intrinsic inflammatory response of upcyte^®^ hepatocytes upon GO treatment in the absence of specialized, non-parenchymal pro-inflammatory cells (i.e., Kupffer cells). We found none of the four tested mediators was altered at the experimental conditions applied in this study. However, by these preliminary data, we cannot exclude that GO may induce inflammation *in vivo*, where the inflammatory cascade can be triggered by other players, which, in turn, are absent in our experimental setting. In this regard, current literature data show that an intravenous injection of GO of different sizes (small-GO, range 50–200 nm; medium-GO, range 200–500 nm; and large GO, range 500–2000 nm) in an IL-6 reporter mouse model produced in parenchymal liver cells an IL-6 activation, which was directly dependent on GO concentration and size (Zhang et al., 2020). In parallel, enhanced IL-6 gene expression and secretion was confirmed *in vitro* using different hepatic cell lines (Hepa1-6, HepG2, and Huh7 cells) (Zhang et al., 2020). A weak pro-inflammatory response was also detected in a 3D airway model (primary human bronchial epithelium), sub-chronically treated with increasing concentration of aerosolized GO (Di Cristo et al., 2020). Nonetheless, though a pro-inflammatory effect induced by GO is evidenced, contradictory data are emerging. Indeed, Lee et al. demonstrated *in vivo* that GO polarized invariant natural killer T-cells towards an anti-inflammatory phenotype, dampening pro-inflammatory cytokine production, and protecting mice against liver injuries due to induced inflammatory responses (Lee et al., 2018). Similarly, Han et al. reported that GO attenuated inflammation by modulating the polarization of mouse macrophages (Han et al., 2018). Taken together such information, further investigations are required for elucidating the inflammatory response induced by GO; however, the role of the immune systems and relative mediators released in the liver microenvironment must be considered as a key aspect if the focus is the cell inflammation response.

In the second part of this study, we focused on two main hepatocyte-specific functions, such as xenobiotics metabolism/transport and acute-phase reaction activation, taking advantage of the peculiar features of upcyte^®^ hepatocytes. Regarding the metabolism of xenobiotics/drugs, cytochromes P450 represent the most important phase-I enzymes in human hepatocytes (Anzenbacher and Anzenbacherová 2001). In the presented study, we selected as potential GO targets the isoforms CYP3A4, CYP2C9, CYP2B6, and CYP1A2, which are the most representative enzymes involved in biotransformation reactions of the liver (Achour et al., 2014). Results show that, at sub-lethal doses, GO dose-dependently downregulated all tested CYPs. For CYP3A4 and CYP2C9, the effect of inhibition was also demonstrated for the enzyme metabolic activities, suggesting either a direct inhibitory effect of GO or a reduced amount of such enzymes caused by the reduced corresponding transcription. Such findings reinforced the preliminary evidence of GO inhibitory effect on cytochrome P450 system reported by Strojny et al. (Strojny et al., 2018). In that work, although using two different *in vitro* approaches (such as the differentiated HepaRG™ cells for monitoring CYP gene expression and a microsomal-based model for assessing CYP metabolic activity), it has been reported a strong reduction of transcription of many CYP isoforms (CYP3A4, CYP2B6, CYP1A2, and CYP2E1) and the inhibition of the catalytic activity of CYP3A4, CYP1A2, and CYP2D6 by GO (Strojny et al., 2018). Similarly, Sekretarska et al. showed, using a microsomal-based model, that GO (50 and 100 μg/ml) caused inhibition of CYP2C9 catalytic activity higher than 40% compared to the control, but, in HepG2 cells, GO did not affect the relative gene expression (Sekretarska et al., 2019). It is worthwhile mentioning that the presented data on CYP gene expression and metabolic activity were obtained using the same cell model and, hence, provided for the first time a complete description of the CYP-inhibition effect of GO in functional, differentiated hepatocytes. Continuing our investigations, we found that GST (phase-II enzyme) and ABCG2 (phase-III efflux transporter) were not affected by acute, sub-lethal GO exposure, suggesting that GO could interfere with the body’s xenobiotic detoxification function, preferentially at the level of phase I system, in our cell model.

Inflammation and its mediators *in vivo* determine the inhibition of CYPs via a multitude of complex pre- and post-transcriptional mechanisms, which, among many others, involve the repression of nuclear factors (such as PXR and CAR), and the modulation of APPs via IL-6 (de Jong et al., 2020; Stanke-Labesque et al., 2020; Lenoir et al., 2021). However, at the experimental conditions applied in our study (i.e., sub-lethal doses), GO did not stimulate an inflammatory response in our cell model (no statistical upregulation of TNFα, IL-1β, IL-6, and IL-8 in GO-treated cells with respect to the control). Hence, we hypothesize that GO may display an intrinsic pro-inflammatory behaviour, provoking the impairment of the cytochrome P450 system acting at different levels of the inflammatory signalling cascade (for instance, suppressing CYPs transcriptional regulators and/or altering the production of different APPs). Notably, Strojny et al. reported that in HepaRG™ cells GO (50 μg/ml) significantly downregulated CAR and PXR (Strojny et al., 2018), which are nuclear receptors acting as ligand (xenobiotic)-dependent, transcription regulators of a large part of phase-I, -II and -III executioners (Evans and Mangelsdorf 2014). Analogously, using PHH as hepatic cell model, it has been reported GO (20 μg/ml) decreased PXR protein (Ženata et al., 2020). In our cell model, we confirmed the GO-induced downregulation of PXR only by the highest concentration (80 μg/ml), while CAR resulted unaffected. Overall, except for weak differences due to the experimental conditions, all these data confirm that PXR can be altered by GO cellular exposure. This finding agrees with recent *in vivo* findings showing that nano-copper-treated rats display a clear PXR downregulation with a concomitant CYP suppression (Tang et al., 2018).

Furthermore, our study showed that the gene expression of CYP3A4 was early downregulated by GO (3 and 8 h) with the maximal effect after 24 h of treatment. Concomitantly, the gene expression of CRP, which is a clinically relevant early and positive APP (Gabay and Kushner 1999), was statistically upregulated by GO starting from 3 h of treatment, with a maximal peak at 8 h. At the same time, an increased secretion of CRP was found. These data show that a peak of CRP occurred earlier than the maximal downregulation of CYP3A4, defining a temporal pattern of gene modulation by GO. Interestingly, in our cell model, the 8-h treatment with the positive control rhIL-6 (the chief regulator of APP synthesis in human hepatocytes (Castell et al., 1989)) determined a production of CRP coupled with the downregulation of CYP3A4. Such effect was comparable to that obtained upon GO treatment. In this regard, Jover et al. pointed to a common transducing step (gp130 receptor) between CYP3A4 downregulation and APPs upregulation by IL-6 in human BC2 cells and also disclosed the molecular mechanism underlying the downregulation of CYP3A4 by IL-6 (Jover et al., 2002). Our data indicate that GO itself could act as an IL-6-type cytokine in stimulating the CRP. Interestingly, a dose-dependent reduction of both Albumin gene expression and translation was also observed in our cell model. Being Albumin considered a “negative” APP (Castell et al., 1989; Gabay and Kushner 1999), this finding further strengthens the evidence that the GO-induced impairment of CYP3A4 is coupled with the activation of acute-phase response. A further confirmation was provided by the downregulation of Transferrin and Transthyretin, which are as Albumin “negative” APPs, downregulated during an acute phase response by the liver (Gabay and Kushner 1999). Regarding Albumin, in particular, after its production and secretion from hepatocytes, enters the bloodstream and regulates plasma colloid osmotic pressure (Chojkier 2005; Merlot et al., 2014). Moreover, this protein can bind and transport ions and numerous endogenous and exogenous substances, including drugs. Consequently, Albumin is involved in the metabolism of transported substances, such as the distribution and the pharmacokinetics of administered drugs. In relation to these functions, a possible GO-induced hypoalbuminemia could be clinically relevant because if this effect was confirmed *in vivo*, it would be related to many adverse effects such as deficits of nutrients or altered drug pharmacokinetics but also oedemas and ascites due to excessive accumulation of fluids (Chojkier 2005; Gatta et al., 2012). Noteworthy, our *in vitro* data obtained using human hepatocytes match well with recent *in vivo* findings. Bengtson et al. reported in GO exposed mice an early and transient upregulation of pulmonary and hepatic APPs (SAA3 and SAA1, respectively), along with an increased systemic level of SAA3 (Bengtson et al., 2017). In addition, also the transcriptome profiling in mice exposed to GO revealed that, in the lung, the APP family was among the top regulated pathways by GO exposure (Poulsen et al., 2021). Overall, the impairment of the cytochrome P450 system caused by GO exposure could have severe consequences for human health, such as impaired detoxification from xenobiotics and drugs, with an increased risk of adverse side effects.

## Conclusion

**FIGURE 8:**
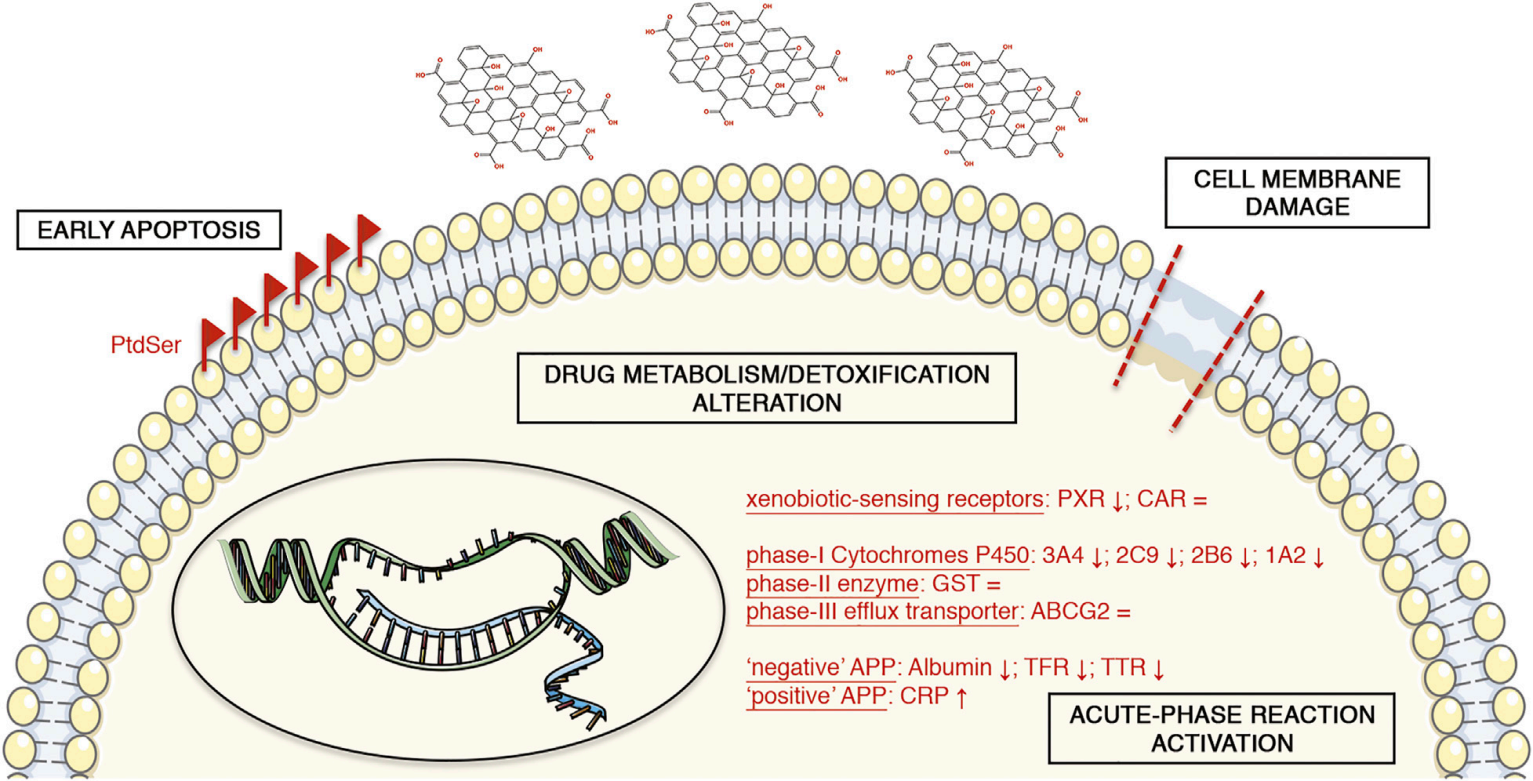
Schematic representation of the GO-induced toxicity in upcyte^®^ hepatocytes. The symbols “↑” and “↓” mean the positive or negative gene expression regulation of the corresponding marker, respectively. The symbol “ = ” indicates no alterations of the corresponding marker. The illustrations of the cell components are from smart.servier.com (published by LES LABORATOIRES SERVIER, SAS).

In conclusion, we investigated the effects of GO upon acute stimulation in upcyte^®^ hepatocytes. Taking advantage of the peculiar features of these cells, we could assess in only one cell model various cell responses to GO, such as cytotoxicity and stress-related responses (apoptosis, oxidative stress, and inflammatory response), along with hepatocyte-specific functions. This broad description of the GO impact on the hepatic tissue is a novel approach for assessing nanomaterial-induced hepatotoxicity. Although some findings in this direction have already been reported in literature, they derive from a combination of different cellular and acellular models (e.g., established cell lines and/or microsomal-based *in vitro* assays). The upcyte^®^ hepatocytes allowed us to study reliably the response of representative cytochromes P450 (such as CYP3A4, CYP2C9, CYP2B6, and CYP1A2) in terms of gene expression and/or metabolic activity, along with other hepatocyte-specific markers (phase-II GST, phase-III ABCG2, and APPs). Our data indicate that GO may induce cell membrane damage and an early stage of apoptosis, along with an alteration of the physiological hepatic functionality (Figure 8). We show that GO may interfere with the body’s drug metabolism/detoxification function, preferentially at the level of the phase-I cytochrome-P450 system via the activation of an acute phase response. This impact is clinically relevant because an altered metabolism of drugs can contribute significantly to the variability of drug responses and could implicate an increased risk of adverse effects, along with altered detoxification. Moreover, the GO impact on Albumin production could determine hypoalbuminemia with a consequent alteration of drug distribution and pharmacokinetics. In addition, we also showed that the activation of an acute-phase reaction and, in particular, the release of CRP is related with the CYP3A4 impairment. These results match well with recent *in vivo* data. Consequently, the presented mechanism of action underlying GO hepatotoxicity may pave the way for developing an *in vitro* platform suitable for screening and bio-monitoring of NP exposure to humans. The findings of this study open up many important questions as, for instance, the role of immune system cells, which, being also resident in liver sinusoids (i.e., Kupffer cells), can remove xenobiotics such as nanomaterials as well as activate the inflammatory cascade (Tsoi et al., 2016; Zhang et al., 2016). For these reasons, more complex *in vitro* models (e.g., co-cultures or liver organoids) and, ultimately, *in vivo* studies could be beneficial to clarify the GO clearance by the liver and the effective hepatotoxicity of GO.

## Methods

### Materials

Second-generation upcyte^®^ hepatocytes (donor 653-03) and Hepatocyte High Performance Medium (complete HHPM: basal medium supplemented with supplement A and L-glutamine) were purchased from upcyte^®^ technologies GmbH (Hamburg, DE). Cell culture flasks and 96- and 24-well plates were from Corning Incorporated (Corning, NY, United States). Collagen Type I solution from rat tail, Rifampicin, Ciprofloxacin, high glucose phenol red-free Dulbecco’s Modified Eagle’s Medium (DMEM), Dulbecco’s Phosphate Buffered Saline (DPBS), resazurin sodium salt, 7-benzyloxy-4-trifluoromethylcoumarin, 7-methoxy-4-trifluoromethylcoumarin, *β*-glucuronidase/arylsulfatase, Lipopolysaccharide (LPS), recombinant human Interferon gamma (rhIFNγ), Triton X-100 and Staurosporine (Stau) were from Sigma Aldrich (Merck KGaA, Darmstadt, DE). Tecan Spark^®^ multimode microplate reader was from Tecan (Männedorf, CH). Recombinant human Interleukin 6 (rhIL-6) and Human CRP Quantikine^®^ ELISA kit were from R&D Systems (Bio-Techne, Minneapolis, MN, United States). CytoTox96^®^ Non-Radioactive Cytotoxicity Assay and RealTime-Glo™ Annexin V Apoptosis and Necrosis Assay were purchased from Promega Corporation (Madison, WI, United States). Prism software was from GraphPad Software (San Diego, CA, United States). Zetasizer Nano-ZS was from Malvern Instruments (Worcestershire, United Kingdom). Pierce™ BCA Protein Assay Kit, TBS Buffer and primary antibody anti-cleaved-PARP were obtained from Thermo Fisher Scientific (Waltham, MA, United States). TRIzol™ Reagent, SuperScript™ VILO™ cDNA Synthesis Kit, qPCR primers and NuPAGE™ 4–12% Bis-Tris gel were obtained from Invitrogen (Thermo Fisher Scientific, Waltham, MA, United States). NanoDrop One^C^ was from Thermo Scientific™ (Thermo Fisher Scientific, Waltham, MA, United States). iTaq™ Universal SYBR^®^ Green Supermix, Clarity™ Western ECL Substrate and ChemiDoc™ MP Imaging System were from Bio-Rad (Hercules, CA, United States). Applied Biosystems ViiA 7 Real-Time PCR System was from Life Technologies (Thermo Fisher Scientific, Waltham, MA, United States). Amersham™ Protran™ 0.2 μm NC nitrocellulose blotting membrane was from GE Healthcare (Buckinghamshire, United Kingdom). Primary antibodies anti-HO-1, anti-SOD1, anti-Albumin and anti-β-Actin, and secondary anti-rabbit and anti-mouse HRP-linked antibodies were purchased from Cell Signaling Technology (Danvers, MA, United States). The illustrations of the cell components are from smart.servier.com (LES LABORATOIRES SERVIER, SAS, Suresnes, FR).

### Graphene Oxide Synthesis and Characterization

Graphene oxide (GO) was kindly provided by Dr E. Vazquez (Universidad de Castilla-La Mancha, Spain). Characterization of the nanomaterial dispersed in Milli-Q^®^ water was previously provided by our group (Guarnieri et al., 2018; Di Cristo et al., 2020). GO colloidal stability was analysed as a function of concentration (4, 20, and 80 μg/ml) and incubation time (0, 0.5, 1.5, 2, 3, 6, and 24 h at 37°C) when dispersed in Milli-Q^®^ water or complete HHPM. GO size distribution profiles were determined via dynamic light scattering (DLS) analysis using a Zetasizer Nano-ZS at 37°C, even though such technique is not entirely accurate for the study of non-spherical particles. Each suspension was prepared directly into a cap-closed disposable cuvette for DLS measurements and incubated at 37°C with no further transfers in order to replicate the same conditions of biological experiments. Three consecutive measurements, with 7 runs per measurement, were performed for each GO suspension. The attenuator and the optimal measurement position were set automatically. GO-free complete HHPM was used as background control. For aqueous suspensions, GO surface charge was measured via Zeta Potential (ZP) analysis using a Zetasizer Nano-ZS at 25°C. Five consecutive measurements, with an automatically set number of runs per measurement, were taken for each GO suspension. To study particle-corona complexes, at the end of 24-h incubation in complete HHPM, GO suspensions were centrifuged at 15 000 g for 15 min at 4°C, and corresponding pellets were washed three times, adding a volume of Milli-Q^®^ water equal to the initial volume (Maiorano et al., 2010; Magrì et al., 2018). Particle-corona complexes were characterized via DLS and Zeta Potential, as described above. GO was endotoxin-free, as previously reported by Di Cristo et al. (Di Cristo et al., 2020), in accordance with US Food and Drug Administration guidelines (https://www.fda.gov/regulatory-information/search-fda-guidance-documents/guidance-industry-pyrogen-and-endotoxins-testing-questions-and-answers).

### Upcyte^®^ Hepatocyte Culture

Second-generation human upcyte^®^ hepatocytes from a female Caucasian donor (653-03) were cultured following the manufacturer’s indications. Upcyte^®^ hepatocytes were seeded at the concentration of 10 000 cells/cm^2^ into cell culture flasks coated with 0.1 ml/cm^2^ of 50 μg/ml collagen-type I in 20 mM acetic acid, and they were cultured in complete HHPM, in incubation in a humidified atmosphere at 37°C, with 5% CO_2_. No antibiotics were added to the culture medium to do not alter cytochromes P450 activity. The culture medium was changed 3 times per week. Cells were expanded for 1 or 2 passages before being treated, as described below.

### Cell Viability Assay

The cell viability upon GO treatment was evaluated by colorimetric resazurin reduction test. Upcyte^®^ hepatocytes were cultured into collagen-coated, flat-bottom 96-well plates (cell growth area equal to 0.3 cm^2^, approximately) and, at the confluence, they were treated with different GO concentrations (final volume equal to 75 μL per well) for 24 h. A 24-h treatment with the complete medium supplemented with 0.03% Triton X-100 was used as a positive control of cell viability reduction. At the end of GO treatment, stimulation media were replaced with 100 μL per well of serum-free phenol red-free high glucose DMEM, supplemented with 44 μM resazurin sodium salt, after having extensively washed with DPBS (O'Brien et al., 2000). After 1 h of incubation at 37°C in a humidified atmosphere with 5% CO_2_ in the dark, the resazurin solution was transferred into a clean 96-well plate, and fluorescence was measured at 535 nm by Tecan Spark^®^ reader. For each culture condition, three independent experiments were performed, each one with a technical triplicate. In each experiment, a couple of cell-free, collagen-coated wells was incubated with the stimulation medium per culture condition (containing GO at all the concentration tested) and used as blank value to be subtracted during the data analysis and to exclude any GO induced optical interferences. The reported results are expressed as percentage values (means ± SD) compared to the control condition (set as 100%). The half maximal inhibitory concentration (IC_50_) of GO was calculated using the log (inhibitor) vs. normalized response curves model on Prism software.

### Cytotoxicity Assay

For evaluating the cell membrane damage upon GO treatment, the colorimetric CytoTox96^®^ Non-Radioactive Cytotoxicity Assay was used. Upcyte^®^ hepatocytes were cultured into collagen-coated, flat-bottom 96-well plates (cell growth area equal to 0.3 cm^2^, approximately). At the confluence, cells were treated with different GO concentrations (final volume equal to 75 μL per well) for 24 h. As a positive control, confluent cells were treated with the complete medium supplemented with 0.03% Triton X-100 for 24 h. At the end of stimulation, conditioned media were collected, centrifuged at 15 000 g for 15 min at 4°C, and analysed following the manufacturer’s instructions. For each culture condition, three independent experiments were performed, each one with a technical triplicate. In each experiment, a couple of cell-free, collagen-coated wells was incubated with the stimulation medium per culture condition (containing GO at all the concentration tested) and used as blank value to be subtracted during the data analysis and to exclude any GO induced optical interferences. The results are expressed as a percentage net increase of the absorbance (means ± SD) compared to the positive control (set as 100%) after having subtracted the basal value of the control condition (set as 0%).

### Apoptosis and Necrosis Assay

For assessing the induction of apoptosis and necrosis, the RealTime-Glo™ Annexin V Apoptosis and Necrosis Assay was used, according to the manufacturer’s instructions. Upcyte^®^ hepatocytes were cultured into collagen-coated, flat-bottom 96-well plates (cell growth area equal to 0.3 cm^2^, approximately). At the confluence, cells were treated for 24 h with the complete medium supplemented with GO at different concentrations, in the presence of the detection reagent (final volume equal to 75 μL per well). As a positive control, confluent cells were treated for 24 h with 0.5 μM Staurosporine (Stau), diluted in the same incubation medium. After 3, 6, and 24 h of incubation, luminescence and fluorescence (at 530 nm) were measured by Tecan Spark^®^ reader for the induction of apoptosis and necrosis, respectively. For each culture condition, three independent experiments were performed, each one with a technical triplicate. In each experiment, a couple of cell-free, collagen-coated wells was incubated with the stimulation medium per culture condition (containing GO at all the concentration tested) and used as blank value to be subtracted during the data analysis and to exclude any GO induced optical interferences. Apoptosis data are expressed as an n-fold increase over the control conditions (means ± SD) for each incubation time. Necrosis results are expressed as net increase of fluorescence for each condition (means ± SD), after having subtracted the control cells’ basal value obtained after 3 h of incubation (set as 0 a.u.).

### Reverse Transcription and Quantitative Real-Time PCR

For the gene expression analysis of GO-treated cells, upcyte^®^ hepatocytes were cultured into collagen-coated, flat-bottom 24-well plates (cell growth area equal to 2.0 cm^2^, approximately) and, at the confluence, they were treated with different GO concentrations (final volume equal to 500 μL per well) for 24 h. Cells treated with 400 μM H_2_O_2_ overnight were used as positive control for the oxidative stress. Regarding the inflammatory response, a 24-h treatment with 1 μg/ml LPS and 50 ng/ml rhIFNγ was used for the upregulation of TNFα, while 100 μg/ml LPS overnight was used for the upregulation of IL-1β, IL-6, and IL-8. As positive and negative controls of CYP3A4/2C9 induction, confluent cells were treated for 72 h with 50 μM Rifampicin or 100 μM Ciprofloxacin, respectively, changing the stimulation medium every day after an extensive wash with DPBS (Bjornsson et al., 2003; Fahmi et al., 2008). For studying the gene expression of CYP3A4 and CRP over time, upcyte^®^ hepatocytes at P3 were cultured into collagen-coated 24-well plates (65 000 cells/cm^2^) for 1 day and, then, treated with 4 μg/ml GO (500 μL/well) for 3, 8 and 24 h. As positive control, cells were treated with 20 ng/ml rhIL-6 (specific activity equal to about 1.1 × 10^5^ IU/μg) for 8 h. At the end of any stimulation, cells were extensively washed with DPBS and incubated with TRIzol™ Reagent (500 µL per well) at −80°C for at least one night. For each sample, total RNA was isolated according to Chomczynski’s method (Chomczynski 1993). RNA yield was determined using NanoDrop One^C^, and RNA purity was examined considering A260/A280 and A260/A230 ratios. Total RNA (2 μg/sample in 20 µL of total volume reaction) was reverse-transcribed to first-strand cDNA by SuperScript™ VILO™ cDNA Synthesis Kit, following manufacturer’s instructions. Transcript levels of target genes were measured by quantitative Real-Time PCR (qPCR) using iTaq™ Universal SYBR^®^ Green Supermix on Applied Biosystems ViiA 7 Real-Time PCR System. Primer sequences, annealing temperatures, corresponding amplicon sizes, and qPCR efficiencies are reported in Supplementary Table S2. The qPCR efficiency of each primer pair was obtained using the dilution model and calculated according to the equation: E = 10^(−1/slope)^, where “slope” is the linear regression slope of a standard curve (Pfaffl and Dorak 2007). For each primer pair, melting curve analysis was carried out to verify the production of a single amplicon and, consequently, primer specificity. The gene expression of GAPDH was used as endogenous control (reference gene). For assessing the reference gene stability across the samples, the n-fold changes in GAPDH transcript level were calculated as E^−ΔCq^, where Cq refers to the quantification cycles, for all samples versus the control condition and ordinary one-way ANOVA was run on those transcript fold changes, obtaining no significant differences (*p* = 0.6161; *n* = 3) (Sundaram et al., 2019). The target transcript levels were calculated using Pfaffl’s model for relative quantification (Pfaffl 2001). For each sample, the reaction was performed in technical triplicate. The results are expressed as the average of at least three independent experiments, with relative SD values.

### Western Blot

For evaluating GO effects on apoptosis and oxidative stress, the protein content from GO-treated upcyte^®^ hepatocytes was analysed by western blot. In particular, proteins were isolated starting from the same homogenates used for RNA isolation as described by Chomczynski (Chomczynski 1993). The protein content was quantified by Pierce™ BCA Protein Assay Kit, following the manufacturer’s instructions. Electrophoresis was performed loading 20 μg of total protein per sample on NuPAGE™ 4–12% Bis-Tris gel under reducing conditions. Proteins were transferred to an Amersham™ Protran™ 0.2 μm NC nitrocellulose blotting membrane. The blot was blocked with 5% non-fat milk in TBS Buffer, supplemented with 0.1% Tween 20 (T-TBS), for at least 1 h at room temperature and then probed with primary antibodies raised against cleaved-PARP (1:1,000), HO-1 (1:1,000), SOD1 (1:500), Albumin (1:1,000) or *β*-Actin (1:1,000), overnight in a cold room. *β*-Actin was considered as the internal control. The blot was extensively washed with T-TBS and incubated with secondary anti-rabbit (1:15,000) or anti-mouse (1:10,000) HRP-linked IgG antibodies for 1 h at room temperature. The blot was then washed with T-TBS and incubated with Clarity™ Western ECL Substrate, following the manufacturer’s instructions. The blot image was acquired by ChemiDoc™ MP Imaging System. Each considered marker was analysed by western blot in three independent experiments. The densitometric analysis was performed by quantifying band densities by Fiji software (http://fiji.sc (Schindelin et al., 2012)). The reported results are expressed as n-fold changes (means ± SD) over the control condition (set as 1.0).

### Cytochrome P450 Activity Assay

For evaluating the enzymatic activity of CYP3A4 and CYP2C9 in GO-treated cells, the conversion of 7-benzyloxy-4-trifluoromethylcoumarin (BFC) or 7-methoxy-4-trifluoromethylcoumarin (MFC) to 7-hydroxy-4-trifluoromethylcoumarin (HFC) was monitored as described by Donato et al. (Donato et al., 2004), with some modifications. Upcyte^®^ hepatocytes were seeded into collagen-coated, flat-bottom 96-well plates (cell growth area equal to 0.3 cm^2^, approximately) and, at the confluence, they were treated with different GO concentrations for 24 h (final volume equal to 75 μL per well). As positive and negative controls, confluent cells were treated for 72 h with complete medium supplemented with 50 μM Rifampicin or 100 μM Ciprofloxacin, respectively, changing the stimulation medium every day after an extensive wash with DPBS (Bjornsson et al., 2003; Fahmi et al., 2008). At the end of treatments, cells were washed with DPBS, and they were incubated with 100 μL per well of 100 μM BFC or 150 μM MFC in the incubation medium (1 mM Na_2_HPO_4_, 137 mM NaCl, 5 mM KCl, 0.5 mM MgCl_2_, 2 mM CaCl_2_, 10 mM glucose, 10 mM Hepes; pH 7.4 buffered solution) for 2 h at 37°C, in a humidified atmosphere with 5% CO_2_. After the incubation with BFC or MFC, the supernatant was collected, diluted 1:1 (v/v) with *β*-glucuronidase/arylsulfatase (150 Fishman units/mL and 1,200 Roy units/mL, respectively) and incubated for 2 h at 37°C. At the end of this step, the reaction mixture was diluted 1:1 (v/v) with the quenching solution (0.25 M Tris in 60% acetonitrile). Finally, the fluorescent HFC metabolite formation was quantified at the wavelength of 410 nm (excitation) and 510 nm (emission) by Tecan Spark^®^ reader. Each culture condition was assayed in technical triplicate in three independent experiments. In each experiment, a couple of cell-free, collagen-coated wells was incubated with the stimulation medium per culture condition (containing GO at all the concentration tested) and used as blank value to be subtracted during the data analysis and to exclude any GO induced optical interferences. The reported results are expressed as percentage values (means ± SD) over the control condition (set as 100%).

### C-Reactive Protein Quantitative Determination Assay

For determining the secretion of CRP into the cell supernatants, the Human CRP Quantikine^®^ ELISA kit was used. At the end of the 8-h incubation, the stimulation media derived from the corresponding qPCR experiments were saved, centrifuged at 15,000 g for 15 min at 4°C and analysed according to manufacturer’s instructions. The cell supernatants derived from cells treated with 20 ng/ml rhIL-6 for 8 h served as positive control of CRP secretion. For each culture condition, four independent experiments were performed. Results are expressed as n-fold changes (means ± SD) over the control condition (set as 1.0).

### Statistical Analysis

Statistical analysis was run on Prism software. Ordinary one-way ANOVA was performed for cell viability assay, cytotoxicity assay, apoptosis assay, qPCR analysis, western blot analysis, and cytochrome P450 activity assay. If ANOVA detected statistically significant differences within the data set, Dunnett’s multiple comparisons test (cytotoxicity assay, apoptosis assay, qPCR analysis, and cytochrome P450 activity assay) or Tukey’s multiple comparisons test (cell viability assay) were used to calculate significant differences. Two-way ANOVA was used for necrosis assay, with Sidak’s multiple comparisons test. Unpaired *t*-Test was used to calculate significant differences between the control condition and the drug-treated cells within data sets obtained by qPCR analysis or cytochrome P450 activity assay, and between the control condition and the GO- or rhIL-6-treated cells in CRP quantitative determinations. GO IC_50_ was calculated using the log (inhibitor) vs. normalized response curves model. Correlation between cell viability and cytotoxicity data and between relative gene expression and metabolic activity data of CYP3A4 and CYP2C9 was calculated using a linear regression. All tests were run setting a confidence interval of 95%. When *p* < 0.05, differences were considered statistically significant. All data are presented as means ± standard deviations (SD) of at least three independent experiments.

## Data Availability

The original contributions presented in the study are included in the article/Supplementary Material, further inquiries can be directed to the corresponding author.

## Abbreviations

GO, Graphene Oxide; PHH, Primary Human Hepatocytes; CYP450, Cytochromes P450; OSM, Oncostatin M; NPs, nanoparticles; HPV, Human Papilloma Virus; HHPM, Hepatocyte High Performance Medium; DMEM, Dulbecco’s Modified Eagle’s Medium; DPBS, Dulbecco’s Phosphate Buffered Saline; LPS, Lipopolysaccharide; rhIFNγ, recombinant human Interferon gamma; LDH, Lactate Dehydrogenase; Stau, Staurosporine; PtdSer, Phosphatidylserine; BFC, 7-benzyloxy-4-trifluoromethylcoumarin; MFC, 7-methoxy-4-trifluoromethylcoumarin; HFC, 7-hydroxy-4-trifluoromethylcoumarin; FACS, fluorescence-activated cell sorting; PXR, Pregnane X Receptor; CAR, Constitutive Androstane Receptor; PARP, poly(ADP-ribose) polymerase; HO-1, Heme Oxygenase-1; SOD1, Superoxide Dismutase 1; TNFα, Tumour Necrosis Factor Alfa; IL-1β, Interleukin 1 Beta; IL-6, Interleukin 6; IL-8, Interleukin 8; GST, Glutathione S-Transferase; ABCG2, ATP Binding Cassette Subfamily G Member 2; GLUT2, Glucose Transporter 2; APPs, Acute-Phase Proteins; Acute-Phase Response (APR); CRP, C-Reactive Protein; TGFβ1, Transforming Growth Factor Beta 1; SEM, Scanning Electron Microscopy; TEM, Transmission Electron Microscopy; DLS, Dynamic Light Scattering; ZP, Zeta Potential; IC_50_, half maximal inhibitory concentration. TFR, transferrin; TTR, transthyretin.
