# Supplementary material for: Sub-Lethal Concentrations of Graphene Oxide Trigger Acute-Phase Response and Impairment of Phase-I Xenobiotic Metabolism in Upcyte® Hepatocytes
Source: Front Bioeng Biotechnol. 2022 May 19;10:867728. doi: 10.3389/fbioe.2022.867728 (PMC9161028; doi:10.3389/fbioe.2022.867728)
Supplement: Supplementary file 1 [file DataSheet1.docx]

**Sub-Lethal Concentrations of Graphene Oxide Trigger Acute-Phase Response and Impairment of Phase-I Xenobiotic Metabolism in Upcyte^®^ Hepatocytes**

A. Romaldini^1†^, R. Spanò^1†^, F. Catalano^2^, F. Villa^3^, A. Poggi^3^ and S. Sabella^1*^

^†^ These authors have contributed equally to this work

^1^ D3 PharmaChemistry, Istituto Italiano di Tecnologia, Genoa, Italy

^2^ Electron Microscopy Facility, Istituto Italiano di Tecnologia, Genoa, Italy

^3^ Unit of Molecular Oncology and Angiogenesis, IRCCS Ospedale Policlinico San Martino, Genoa, Italy

^*^ Corresponding author: stefania.sabella@iit.it

**SUPPLEMENTARY METHODS**

**Materials**

JEOL JSM-7500FA, JEOL JSM-6490LA, and JEOL JEM-1011 were from Jeol Ltd. (Akishima, JP); Leica DMI6000 B and related products were purchased from Leica Microsystems (Wetzlar, DE). The 4-chamber culture treated glass slides were from Falcon^®^ (Corning, NY, USA). Prism software was from GraphPad Software (San Diego, CA, USA). Monoclonal anti-GLUT2 antibody was from R&D Systems (Bio-Techne, Minneapolis, MN, USA). Secondary goat anti-mouse antibody conjugated with Alexa Fluor 647 fluorochrome was from Invitrogen (Thermo Fisher Scientific, Waltham, MA, USA). CytoFLEXS flow cytometer and CytExpert 2.8 software were from Beckman Coulter (Brea, CA, USA).

**GO characterization**

GO morphology was evaluated by scanning electron microscopy (SEM), carried out by JEOL JSM-7500FA (high resolution analytical field-emission scanning electron microscope) equipped with a cold field emission gun and with energy-dispersive X-ray spectroscopy (EDS). Transmission electron microscopy (TEM) was also performed using JEOL JEM-1011, equipped with a W thermionic source operating at 100 kV.

**GO deposits characterization by the analysis of optical microscopy images**

Upcyte^®^ hepatocytes were cultured into collagen-coated, flat-bottom 96-well plates (cell growth area equal to 0.3 cm^2^, approximately) and, at the confluence, they were treated with different GO concentrations (final volume equal to 75 μL per well) for 24 hours. For each experimental condition, at the end of GO treatment, representative areas of interest were acquired by optical microscope Leica DMI6000 B equipped with Leica CTR6000 light source and Leica DFC360 FX camera, at the optical magnification of 40x with the objective Leica HCX-PL FLUOTAR L 40x/0.60 CORR PH2. The obtained grayscale images have been analysed by Fiji software (<http://fiji.sc> [1]). A manual threshold was defined for each image to visualize only the GO deposits (black) and a mask was created with this area selection (see **Figure S2C**). The tool ‘Analyze Particles’ was used to measure the total count of objects (*i.e.*, GO deposits) and the percentage area covered by deposits.

**GO deposits characterization by the analysis of SEM images**

Upcyte^®^ hepatocytes were cultured into collagen-coated, 4-chamber culture treated glass slides and, then, they were treated with 4, 20 or 80 μg/mL GO for 24 hours. At the end of GO treatment, cells were fixed with 2% glutaraldehyde in 0.1 M cacodylate buffer for 1.5 hours at room temperature. After having extensively washed with 0.1 M cacodylate buffer, the samples were post-fixed with 1% osmium tetroxide in Milli-Q^®^ water for 2 hours and washed with Milli-Q^®^ water. After that, cells were dehydrated through sequential 10-minutes incubations in rising concentrations of ethanol (from 30 to 100%) and, then, in mixtures of ethanol:hexamethyldisilazane (from 3:1 to 1:3). The samples were air-dried overnight and sputtered with a 10 nm gold layer. For each sample, the GO deposits on the top of treated cells were visualized by scanning electron microscopy (SEM), carried out by JEOL JSM-6490LA (analytical low-vacuum scanning electron microscope) equipped with a tungsten filament and operating at 10 kV of accelerating voltage. The lateral size distributions of GO deposits were calculated on Prism Software, followed by a nonlinear regression (Gaussian model). For each experimental condition, the lateral size distribution was obtained by measuring at least 85 deposits in total.

**FACS analysis**

Upcyte^®^ hepatocytes were cultured into collagen-coated, flat-bottom 24-well plates (cell growth area equal to 2.0 cm^2^, approximately) and, at the confluence, they were treated with 4 or 80 μg/mL GO (final volume equal to 500 μL per well) for 24 hours. At the end of the treatment, cells were detached, counted, and put at the 10^5^ cells/sample for flow cytometry assays. Re-suspended pellets of cells were fixed with 2% (v/v) formaldehyde in DPBS for 20 minutes at room temperature and permeabilized with 70% (v/v) ethanol in distilled water for 30 minutes at 4°C. This procedure allowed the antibody to react better with the corresponding antigen. After that, samples were incubated for 30 minutes at 4°C with anti-GLUT2 monoclonal antibody (at a final concentration of 2.5 µg/mL in culture medium), extensively washed with DPBS, and further incubated for 30 minutes at 4°C with the isotype-specific secondary goat-anti-mouse antibody conjugated with Alexa Fluor 647 fluorochrome. Samples were run on a CytoFLEX flow cytometer (3000 events for each sample) and analyzed with CytExpert 2.4 software.

**SUPPLEMENTARY TABLES**

**Table S1**. Primer pairs used for gene expression analysis by quantitative real-time PCR (qPCR).

| **Target Gene** | **Direction** | **Primer Sequence (5'🡺3')** | **Amplicon Size** | **T_annealing_** | **E_Target_** |
| --- | --- | --- | --- | --- | --- |
| HO-1 | *forward* | CCCACGCCTACACCCGCTAC | 137 bp | 62°C | 2.01 |
|  | *reverse* | GGTGGCACTGGCAATGTTGG |  |  |  |
| SOD1 | *forward* | GGTGTGGCCGATGTGTCTAT | 110 bp | 60°C | 1.85 |
|  | *reverse* | CCTTTGCCCAAGTCATCTGC |  |  |  |
| TNFα | *forward* | TGGGATCATTGCCCTGTGAG | 92 bp | 60°C | 1.89 |
|  | *reverse* | GGTGTCTGAAGGAGGGGGTA |  |  |  |
| IL-1β | *forward* | ATGATGGCTTATTACAGTGGCAA | 132 bp | 58°C | 1.99 |
|  | *reverse* | GTCGGAGATTCGTAGCTGGA |  |  |  |
| IL-6 | *forward* | CCTGAACCTTCCAAAGATGGC | 75 bp | 60°C | 2.10 |
|  | *reverse* | TTCACCAGGCAAGTCTCCTCA |  |  |  |
| IL-8 | *forward* | CCAGGAAGAAACCACCGGA | 91 bp | 58°C | 2.12 |
|  | *reverse* | GAAATCAGGAAGGCTGCCAAG |  |  |  |
| CYP3A4 | *forward* | CCTTACACATACACACCCTTTGGAAGT | 382 bp | 62°C | 1.95 |
|  | *reverse* | AGCTCAATGCATGTACAGAATCCCCGGTTA |  |  |  |
| CYP2C9 | *forward* | ACGGATTTGTGTGGGAGAAGCCC | 287 bp | 62°C | 2.06 |
|  | *reverse* | TGAGATGACAGGTGAGAAAAGGCA |  |  |  |
| CYP2B6 | *forward* | GGGGCACTGAAAAAGACTGA | 118 bp | 58°C | 2.11 |
|  | *reverse* | AGTTCTGGAGGATGGTGGTG |  |  |  |
| CYP1A2 | *forward* | ATGGCATTGTCCCAGTCTGTT | 135 bp | 60°C | 2.00 |
|  | *reverse* | TGGCTCTGGTGGACTTTTCAG |  |  |  |
| GST | *forward* | GATACTGGGGTACTGGGACATCC | 130 bp | 61°C | 1.85 |
|  | *reverse* | CCACTGGCTTCTGTCATAATCAGG |  |  |  |
| ABCG2 | *forward* | CAGGTGGAGGCAAATCTTCGT | 247 bp | 58°C | 2.07 |
|  | *reverse* | ACCCTGTTAATCCGTTCGTTTT |  |  |  |
| PXR | *forward* | CTCACCTCCAGGTTTGCTTC | 114 bp | 55°C | 2.11 |
|  | *reverse* | TCCTTGATCGATCCTTTGC |  |  |  |
| CAR | *forward* | AAGTGCTTAGATGCTGGCATGAGG | 112 bp | 60°C | 2.05 |
|  | *reverse* | CTCAGTTGCACAGGTGTTTGC |  |  |  |
| Albumin | *forward* | CTTGAATGTGCTGATGACAGG | 157 bp | 57°C | 2.06 |
|  | *reverse* | GCAAGTCAGCAGGCATCTCAT |  |  |  |
| CRP | *forward* | TGTGCCTCCACTTCTACACG | 73 bp | 58°C | 1.75 |
|  | *reverse* | TGTCTCTTGGTGGCATACGA |  |  |  |
| Transferrin | *forward* | AACCAATACTTCGGCTACTCGG | 137 bp | 60°C | 2.04 |
|  | *reverse* | AGGCAAAGCAGCTCATACTGG |  |  |  |
| Transthyretin | *forward* | CAGAAAGGCTGCTGATGACAC | 70 bp | 60°C | 1.90 |
|  | *reverse* | ATGCAGCTCTCCAGACTCACT |  |  |  |
| **Reference Gene** | **Direction** | **Primer Sequence (5'🡺3')** | **Amplicon Size** | **T_annealing_** | **E_Ref_** |
| GAPDH | *forward* | AAGGTGAAGGTCGGAGTCAA | 108 bp | 55°C | 2.15 |
|  | *reverse* | AATGAAGGGGTCATTGATGG |  |  |  |

**Notes**. ‘T_annealing_^’^ indicates the annealing temperature of each primer pair. ‘E_Target_^’^ and ‘E_Ref_^’^ refer to qPCR efficiencies for target and reference gene transcripts, respectively, calculated according to E = 10^(-1/slope)^ [2].

**Table S2**. Hydrodynamic diameter of graphene oxide (GO) dispersed in Milli-Q^®^ water and complete HHPM, as a function of concentration and incubation time by means of DLS analysis.

| **Dispersant** | **Incubation** | **[GO]** | **Hydrodynamic Diameter (D_H_)** | | | **Relative Intensity** | | |
| --- | --- | --- | --- | --- | --- | --- | --- | --- |
|  | *hours* | *μg/mL* | *peak #1*  *(nm)* | *peak #2*  *(nm)* | *peak #3*  *(nm)* | *peak #1 (%)* | *peak #2 (%)* | *peak #3 (%)* |
| Milli-Q^®^ water | 0 | **4** | - | 268.7 ± 21.9 | - | - | 100 | - |
|  |  | **20** | - | 306.4 ± 15.0 | - | - | 100 | - |
|  |  | **80** | - | 324.3 ± 11.9 | 5154.3 ± 370.9 | - | 97.2 | 2.8 |
|  | 24 | **4** | - | 267.4 ± 15.8 | - | - | 100 | - |
|  |  | **20** | - | 278.9 ± 2.6 | 5421.0 (n=1) | - | 99.7 | 0.3 |
|  |  | **80** | - | 313.1 ± 16.8 | - | - | 100 | - |
| complete HHPM | 0 | **4** | 7.8 ± 0.4 | 295.0 ± 27.7 | - | 14.9 | 85.1 | - |
|  |  | **20** | - | 338.1 ± 10.0 | - | - | 100 | - |
|  |  | **80** | - | 340.0 ± 38.9 | 4861.0 ± 230.4 | - | 93.6 | 6.4 |
|  | 24 | **4** | 6.0 ± 0.2 | 359.5 ± 1.3 | - | 20.0 | 80.0 | - |
|  |  | **20** | 6.1 ± 0.2 | 505.6 ± 51.7 | - | 14.6 | 85.4 | - |
|  |  | **80** | 5.1 (n=1) | 660.9 ± 55.5 | - | 1.7 | 98.3 | - |

**Notes**. ‘*n = 1’* means the frequency of detection of the corresponding peak, namely, the detection of the peak in only one of three measurements performed. The symbol ‘-‘ means that no peak has been detected in three measurements.

**SUPPLEMENTARY FIGURES**

**Figure S1**


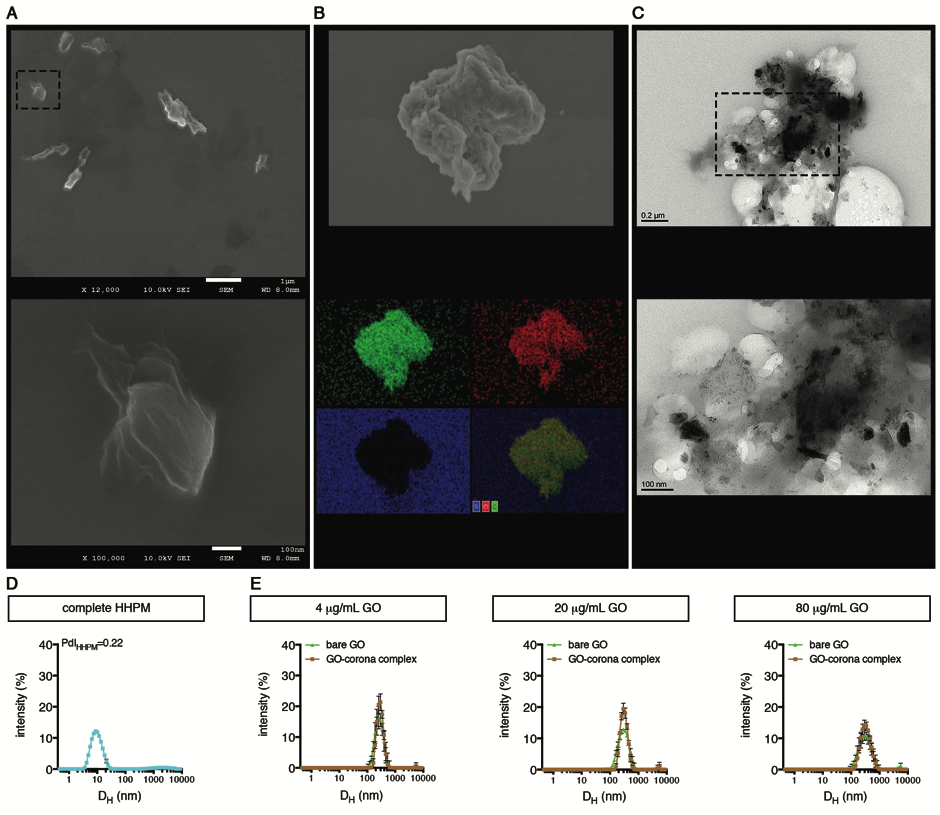


**Figure S1**. (**A**) Representative SEM image of 4 μg/mL GO in Milli-Q^®^ water (12’000x; scale bars = 1 μm). The image below is a magnification of the highlighted area (100’000x; scale bar = 100 nm). (**B**) SEM image and the corresponding elemental map by EDS (below) showing the GO composition, where ‘Si’, ‘O’, and ‘C’ indicate atoms of Silicon, Oxygen and Carbon, respectively. (**C**) Representative TEM image of 4 μg/mL GO in Milli-Q^®^ water (scale bars = 0.2 μm). The image below is a magnification of the highlighted area (scale bar = 100 nm). (**D**) Size distribution profile of complete HHPM obtained via DLS analysis. (**E**) Size distribution profiles by means of DLS analysis of GO dispersed in Milli-Q^®^ water (green curves) and particle-corona complexes derived from the 24-hour incubation of GO in complete HHPM and re-dispersed in Milli-Q^®^ water (brown curves). Measurements refer to three GO concentrations (4, 20, and 80 μg/mL).

**Figure S2**


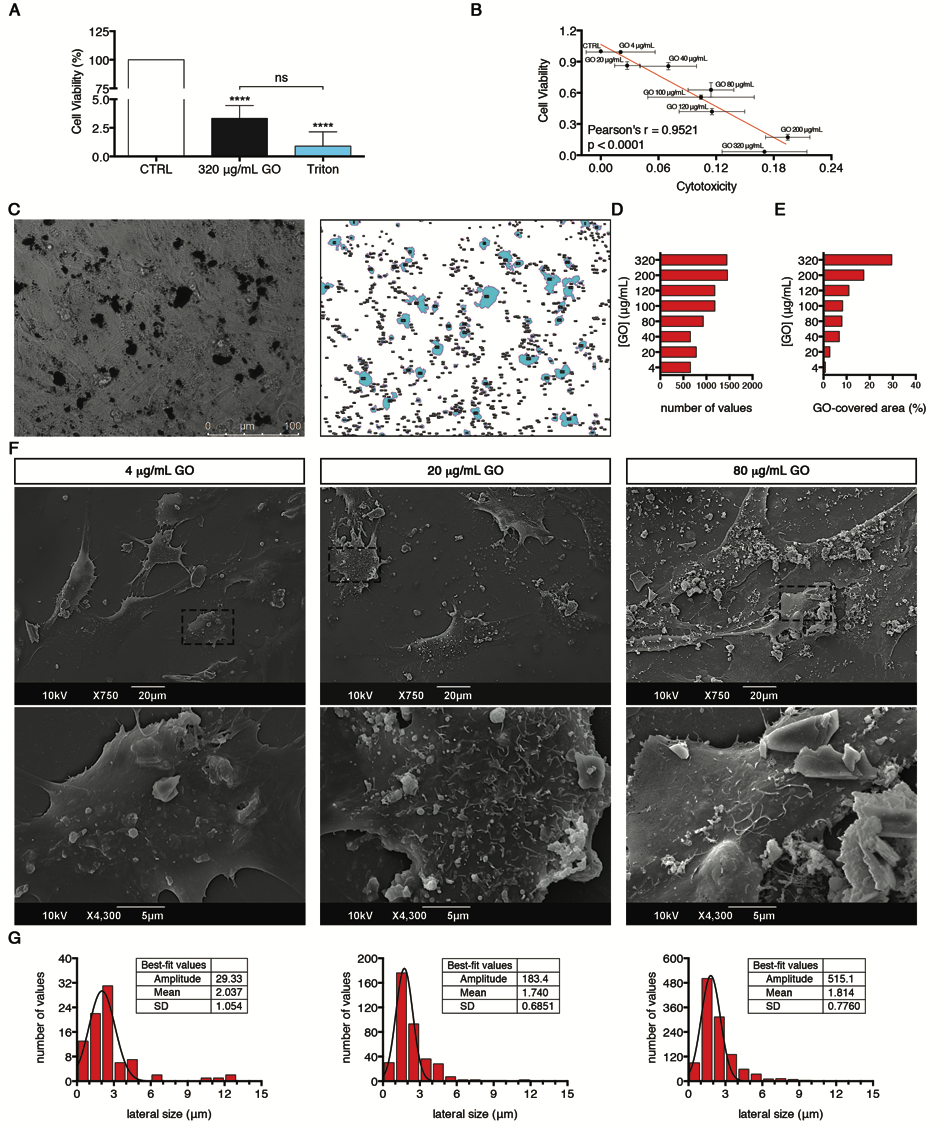


**Figure S2**. (**A**) Cell viability of upcyte^®^ hepatocytes treated for 24 hours with 320 μg/mL GO or with 0.03% Triton X-100 (indicated as ‘Triton’) or un-treated (indicated as ‘CTRL’), evaluated by resazurin reduction assay. Results are expressed as percentage values compared to CTRL (set as 100%). Results represent means ± SD of three independent experiments. The symbol ‘****’ refers to p < 0.0001, calculated with respect to CTRL (ordinary one-way ANOVA). ‘ns’ means there are no statistically significant differences between 320 μg/mL GO and Triton conditions. (**B**) Linear correlation between cell viability and cytotoxicity data (both expressed as fold increase). (**C**) Representative image by optical microscopy of upcyte^®^ hepatocytes exposed to 80 μg/mL GO and the corresponding ‘masked’ image (on the right) obtained creating a specific selection for GO deposits (40x; scale bar = 100 μm). (**D**, **E**) Results calculated on ‘masked’ images relative to GO deposits at the end of 24-hour exposure with 4-320 μg/mL GO. For each experimental condition, the total number of deposits (**D**) and the percentage area covered by deposits (**E**) are shown. (**F**) Representative SEM images of upcyte^®^ hepatocytes treated for 24 hours with 4, 20, and 80 μg/mL GO (750x; scale bars = 20 μm). The images below are magnifications of the highlighted areas (4’300x; scale bar = 5 μm). (**G**) Lateral size distribution relative to GO deposits on the top of cell surface at the end of 24-hour exposure with 4, 20, and 80 μg/mL GO.

**Figure S3**


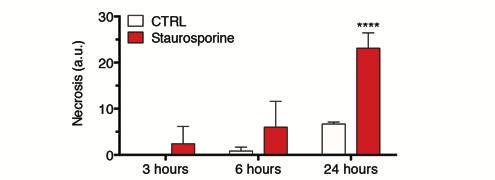


**Figure S3**. Cell necrosis of upcyte^®^ hepatocytes treated for up to 24 hours with 0.5 μM Staurosporine or un-treated (indicated as ‘CTRL’), evaluated by apoptosis and necrosis assay. Results are expressed as a net increase of absorbance for each condition after having subtracted the basal value of CTRL obtained after 3 hours of incubation (set as 0 a.u.). Results represent means ± SD of three independent experiments. The symbol ‘****’ refers to p < 0.0001, calculated with respect to CTRL (two-way ANOVA).

**Figure S4**


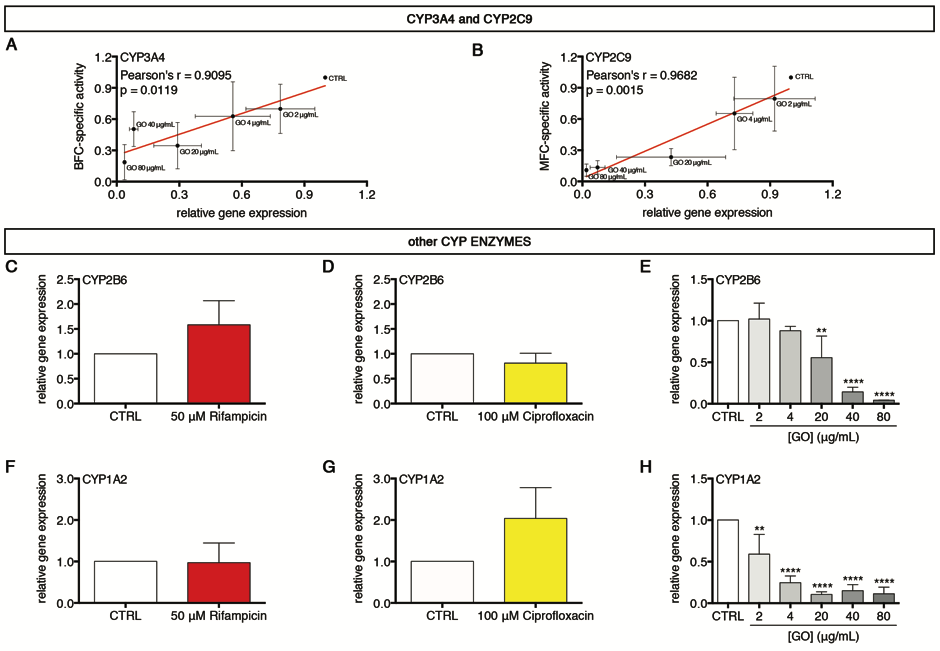


**Figure S4**. (**A**, **B**) Linear correlation between relative gene expression and metabolic activity data (both expressed as n-fold increase with respect to CTRL) for CYP3A4 (**A**) and CYP2C9 (**B**). (**C**, **D**) Relative gene expression of CYP2B6 in cells treated daily, for 72 hours with 50 μM Rifampicin (**C**) or with 100 μM Ciprofloxacin (**D**) *versus* untreated cells (CTRL). Results are expressed as means ± SD values of three independent experiments. (**E**) Relative gene expression of CYP2B6 in cells treated for 24 hours with sub-lethal GO doses *versus* CTRL. Results are expressed as means ± SD values of three independent experiments. The symbols ‘**’ and ‘****’ refer to p = 0.0069 and p < 0.0001, respectively, calculated against CTRL (ordinary one-way ANOVA). (**F**, **G**) Relative gene expression of CYP1A2 in cells treated daily, for 72 hours with 50 μM Rifampicin (**F**) or with 100 μM Ciprofloxacin (**G**) *versus* CTRL. Results are expressed as means ± SD values of three independent experiments. (**H**) Relative gene expression of CYP1A2 in cells treated for 24 hours with sub-lethal GO doses *versus* CTRL. Results are expressed as means ± SD values of three independent experiments. The symbols ‘**’ and ‘****’ refer to p = 0.0052 and p < 0.0001, respectively, calculated against CTRL (ordinary one-way ANOVA).

**Figure S5**


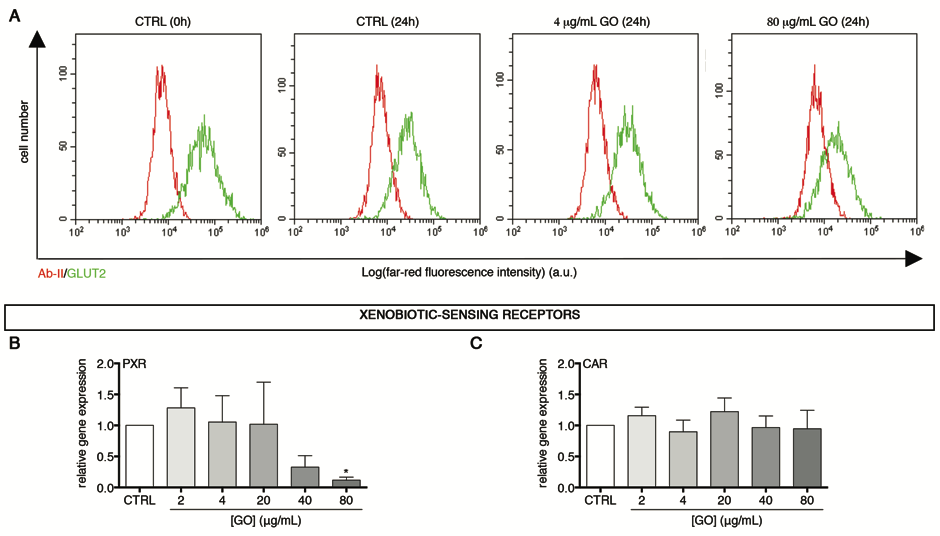


**Figure S5**. (**A**) Expression of GLUT2 in cells before (0h) and after a 24-hour treatment (24h) with sub-lethal GO doses *versus* untreated cells (CTRL), investigated by FACS analysis. In each histogram, results are expressed as Log(far-red fluorescence intensity) (arbitrary unit, a.u.) *versus* cell number. The red curves refer to the cells stained with the second antibody alone, representing the negative control (Ab-II), and the green curves to the cells stained with the anti-GLUT2 antibody. Each panel shows the overlaid signals relative to negative control and GLUT2 expression. (**B**, **C**) Relative gene expression of PXR (**B**) and CAR (**C**) in cells treated for 24 hours with sub-lethal GO doses *versus* untreated cells (CTRL). Results are expressed as means ± SD values of three independent experiments. The symbol ‘*’ refers to p = 0.0224, calculated with respect to CTRL (ordinary one-way ANOVA).

**Figure S6**


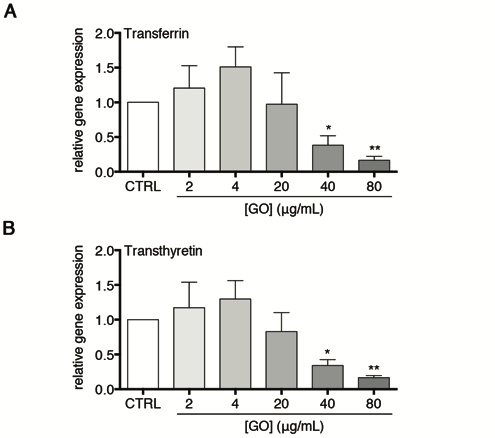


**Figure S6**. (**A**, **B**) Relative gene expression of Transferrin (**A**) and Transthyretin (**B**) in GO-treated cells *versus* untreated cells (indicated as CTRL). Results are expressed as means ± SD values of three independent experiments. The symbols ‘*’ and ‘**’ refer to p ≤ 0.0336 and p ≤ 0.0051, respectively, calculated *versus* CTRL (ordinary one-way ANOVA).

**Figure S7**


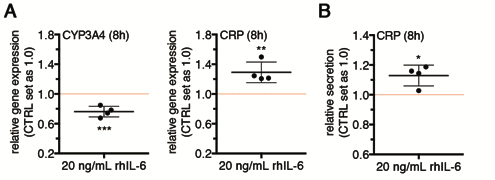


**Figure S7**. (**A**) Relative gene expression of CYP3A4 and CRP in cells treated with 20 ng/mL rhIL-6 *versus* untreated cells (indicated as CTRL) after 8 hours of treatment (indicated as 8h). Results are expressed as mean ± SD value of four independent experiments. The symbols ‘**’ and ‘***’ refer to p = 0.0057 and p = 0.0006, respectively, calculated *versus* CTRL (unpaired t-Test). (**B**) Relative secretion of CRP in rhIL-6-treated cells *versus* CTRL at 8h. Results are expressed as mean ± SD value of four independent experiments. The symbol ‘*’ refers to p = 0.0101, calculated *versus* CTRL (unpaired t-Test).

**REFERENCES**

1. Schindelin J, Arganda-Carreras I, Frise E, Kaynig V, Longair M, Pietzsch T, et al. Fiji: an open-source platform for biological-image analysis. Nat Methods [Internet]. 2012;9:676–82. Available from: http://www.nature.com/articles/nmeth.2019

2. Pfaffl MW. A new mathematical model for relative quantification in real-time RT-PCR. Nucleic Acids Res [Internet]. 2001;29:45e – 45. Available from: https://academic.oup.com/nar/article-lookup/doi/10.1093/nar/29.9.e45
